# Transcriptome Analysis Reveals Key Pathways and Candidate Genes Controlling Seed Development and Size in Ricebean (*Vigna umbellata*)

**DOI:** 10.3389/fgene.2021.791355

**Published:** 2022-01-21

**Authors:** Sachin Kumar Verma, Shikha Mittal, Gayacharan, Dhammaprakash Pandhari Wankhede, Swarup Kumar Parida, Debasis Chattopadhyay, Geeta Prasad, Dwijesh Chandra Mishra, Dinesh Chandra Joshi, Mohar Singh, Kuldeep Singh, Amit Kumar Singh

**Affiliations:** ^1^ ICAR-National Bureau of Plant Genetic Resources, New Delhi, India; ^2^ National Institute of Plant Genome Research, New Delhi, India; ^3^ ICAR- Indian Agricultural Statistics Research Institute, New Delhi, India; ^4^ ICAR-Vivekananda Parvatiya Krishi Anusandhan Sansthan, Almora, India

**Keywords:** ricebean, seed size, hormone signaling, MapMan, SSR

## Abstract

Ricebean (*Vigna umbellata*) is a lesser known pulse with well-recognized potential. Recently, it has emerged as a legume with endowed nutritional potential because of high concentration of quality protein and other vital nutrients in its seeds. However, the genes and pathways involved in regulating seed development and size are not understood in this crop. In our study, we analyzed the transcriptome of two genotypes with contrasting grain size (IC426787: large seeded and IC552985: small seeded) at two different time points, namely, 5 and 10 days post-anthesis (DPA). The bold seeded genotype across the time points (B5_B10) revealed 6,928 differentially expressed genes (DEGs), whereas the small seeded genotype across the time point (S5_S10) contributed to 14,544 DEGs. We have also identified several candidate genes for seed development–related traits like seed size and 100-seed weight. On the basis of similarity search and domain analysis, some candidate genes (*PHO1*, *cytokinin dehydrogenase*, A-type cytokinin, and *ARR* response negative regulator) related to 100-seed weight and seed size showed downregulation in the small seeded genotype. The MapMan and KEGG analysis confirmed that auxin and cytokinin pathways varied in both the contrasting genotypes and can therefore be the regulators of the seed size and other seed development–related traits in ricebeans. A total of 51 genes encoding *SCF* ^*TIR1/AFB*^, *Aux/IAA*, *ARFs*, *E3* ubiquitin transferase enzyme, and *26S* proteasome showing distinct expression dynamics in bold and small genotypes were also identified. We have also validated randomly selected SSR markers in eight accessions of the *Vigna* species (*V. umbellata*: 6; *Vigna radiata*: 1; and *Vigna mungo*: 1). Cross-species transferability pattern of ricebean–derived SSR markers was higher in *V. radiata* (73.08%) than *V. mungo* (50%). To the best of our knowledge, this is the first transcriptomic study conducted in this crop to understand the molecular basis of any trait. It would provide us a comprehensive understanding of the complex transcriptome dynamics during the seed development and gene regulatory mechanism of the seed size determination in ricebeans.

## Introduction

A rapid increase in the human population, which is expected to reach 9.7 billion by 2050, is one of the biggest challenges of this world (Gu et al., 2021). To ensure food and nutritional security to the ever-growing human population, it is extremely important to bring underutilized and neglected crops into mainstream agriculture. Owing to its short growth duration and ability to thrive well in stress conditions and various soil types, ricebean (*Vigna umbellata*) is one such crop which has huge potential to sustain food and nutritional security in most parts of the world (Pattanayak et al., 2019). It is a diploid (2*n* = 2× = 22), warm-season annual legume with a genome size of approximately 440 Mb (Kaul et al., 2019). Ricebean is mainly cultivated in Nepal, Bhutan, Northeast India up to Myanmar, Southern China, Northern Thailand, Laos, Vietnam, Indonesia, and East Timor (Tian et al., 2013), where it constitutes an important source of protein for the sizable population and contributes to household food and nutritional security. The observed protein content in ricebean is 25.57% with high concentration of various essential amino acids. Besides protein, ricebean grains also contain a significant amount of other nutrients such as carbohydrates, fiber, minerals, vitamins, and fatty acids. Moreover, ricebean is a rich source of unsaturated fatty acids like linoleic and linolenic acids (Katoch, 2013).

Among various productivity traits, pod length, seed size, and seed weight have major emphasis on ricebean genetic improvement programs because of their direct impact on the total grain yield. Furthermore, the seed is the key reservoir of proteins, essential amino acids, unsaturated fatty acids, and minerals in ricebean. Therefore, it is of great importance to decipher the molecular mechanism underlying seed development and size determination process in this minor but potential pulse crop. In recent years, with the advent of next-generation sequencing technology, key gene regulatory networks governing pod and seed development have been well characterized in both model plants like rice (Herridge et al., 2011), *Arabidopsis* (Herridge et al., 2011; Mahto et al., 2017), and also in non-model legume species like black gram (Souframanien and Reddy, 2015), cowpea (Lonardi et al., 2019), chickpea (Pradhan et al., 2014), mungbean (Tian et al., 2016a), and soybean (Jones and Vodkin, 2013; Qi et al., 2018; Peng et al., 2021). These studies revealed that seed development in higher plants is a highly complex process and governed by phytohormone signaling including cytokinins (CKs), gibberellins (GAs), brassinolides (BRs), ethylene (ET), and their associated genes and transcription factors. In all these phytohormones, genes related to auxin pathways including indole-3-acetic acid (*IAA*), auxin-responsive protein (*IAA12*, *IAA*), auxin response factors (*ARFs*), *SAUR*-like auxin superfamilies, auxin-related *Aux/IAA*, *OsIAA18*, and *AP2/ERF*, along with other genes such as *ARR-B* (cytokinin signaling), ethylene-responsive transcription factor (*ERF084*-like, *ERF4*, *ERF061*), ethylene-insensitive protein 3 (*EIN3*), ethylene receptor (*ETR*), ethylene-insensitive protein 4 (*EIN4*), serine/threonine-protein kinase (*CTR1*), ethylene responsive *APATELA2* (*AP2*), ethylene-responsive element binding protein (*EREBP*), and many more genes were reported during seed development (Garg et al., 2011; Jones and Vodkin, 2013; Tian et al., 2016a; Nelson et al., 2017; Geng et al., 2018; Li et al., 2019a; Lonardi et al., 2019; Yi et al., 2019; Raizada and Jegadeesan, 2020; Zhu et al., 2020).

The aforementioned transcriptome-based gene expression analysis has provided a robust functional genomics resource for deciphering gene networks and candidate genes regulating various biological processes in crop plants. For minor crops with poorly characterized genomes, like ricebean, such detailed transcriptome analysis will provide comprehensive information about expression patterns of genes and molecular mechanisms governing traits of economic importance. This valuable information can further be employed for the development of functional markers for gene and QTL mapping. Therefore, in the present study, we conducted transcriptome analyses to investigate gene expression networks and identify the candidate genes controlling seed size variation in ricebean. RNA sequencing of two contrasting ricebean genotypes was performed at early development stages (i.e., 5 and 10 DPA). The study provides detailed insights into various gene networks and their potential roles in determining seed size. Furthermore, the study also identified simple sequence repeat (SSR) motifs that could be used for molecular mapping of seed size/weight and other related traits.

## Materials and Methods

### Plant Material and Growth Conditions

**FIGURE 1:**
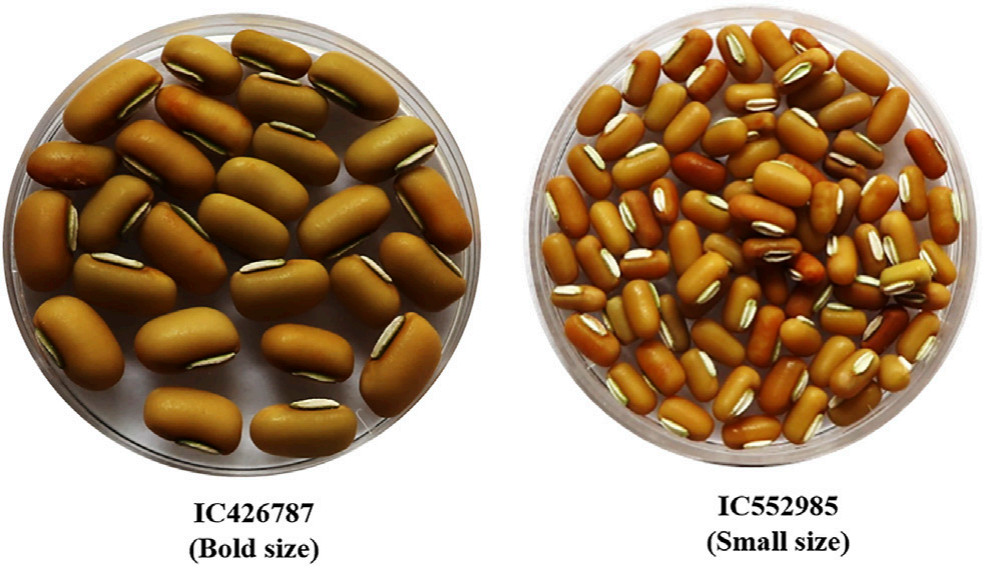
Two contrasting genotypes of ricebean, that is, IC426787 (bold size) and IC552985 (small size), selected for the transcriptome analysis on the basis of their seed size.

Seeds of two contrasting ricebean genotypes, namely, IC426787 (bold seeded) and IC552985 (small seeded) were obtained from ICAR-National Bureau of Plant Genetic Resources (NBPGR), New Delhi (Figure 1). On the basis of the 2-year trial (2018 and 2019), the average 100-seed weight of IC426787 and IC552985 was 13.20 and 3.87 gm, respectively. Plants were grown in a net-house at ICAR-NBPGR, New Delhi (latitude: 28°38′56″N, longitude: 77°9′8″E, altitude: 228 mean sea level (msl)), during *Kharif* (rainy) season 2020. During pod filling, the minimum temperature ranged from 10.8 to 23°C, maximum temperature ranged from 30.4 to 36°C, and average RH% varied from 53 to 56. The ricebean pod filling duration varied from 20 to 30 DPA depending upon the genotype. Genotypes with smaller grain size took comparatively less pod filling time than the genotypes having larger grain size. Three biological replicates of pod samples were harvested from three full-grown plants of both genotypes at 5 and 10 DPA each. The seeds were separated and immediately frozen in liquid nitrogen and stored at −80°C for future use. A total of 12 samples were prepared for the construction of RNA libraries.

### RNA Extraction, Library Preparation, and Sequencing

The Pure Link RNA Mini Kit (Ambion, United States) was used to extract RNA from the frozen samples. The total RNA quality was checked using the RNA 6000 Nano Kit (Agilent Technologies, United States) on a 2100 Bioanalyzer (Agilent Technologies, United States), with a minimum RNA integrity number (RIN) of 7. RNA concentrations were determined with a NanoDrop ND-8000 spectrophotometer (Nano-Drop Technologies, Thermo scientific, Wilmington, DE). RNA-Seq libraries for all samples were prepared using the NEBNext UltraII RNA library preparation kit for Illumina; Cat no: E7770 (New England Biolabs), according to manufacturers recommended protocol, and sequencing was done in a single HiSeq 4000 lane using 150 bp paired-end chemistry. Briefly, total RNA was used to purify poly (A) messenger RNA (mRNA) using oligo-dT labeled magnetic beads. Then, the isolated mRNA was fragmented into 200 to 500 bp pieces in the presence of divalent cations at 94°C for 5 min using an ultrasonicator. The cleaved RNA fragments were copied into first-strand cDNA using SuperScript-II reverse transcriptase (Life Technologies, Inc.) and random primers. After second-strand cDNA synthesis, fragments were end-repaired and A-tailed, and indexed adapters were ligated. The products were purified and enriched with PCR to create the final cDNA library. The tagged cDNA libraries were pooled in equal ratios and used for 2 × 150 bp paired-end sequencing on a single lane of the Illumina HiSeq 4000. Illumina clusters were generated and loaded onto the Illumina Flow Cell on the Illumina HiSeq 4000 instrument, and sequencing was carried out using 2 × 150 bp paired-end chemistry. After sequencing, the samples were demultiplexed, and the indexed adapter sequences were trimmed using CASAVA v1.8.2 software (Illumina Inc.).

### Read Quality and Adapter Removal

Raw reads of ricebean were evaluated for their quality using FASTQC v0.11.8 package (http://www.bioinformatics.bbsrc.ac.uk/projects/fastqc/). Four parameters were considered: base quality score distribution, sequence quality score distribution, average base content per read, and GC distribution in the reads. Trimmomatic v0.36 was applied to remove the adapter and trim the low-quality reads (trimming includes reads with or without ambiguous sequence “N”) using default parameters. To correct the random sequencing errors in Illumina RNA-Seq reads, Rcorrector v1.0.3 was used. Clean reads were also checked for their quality using FASTQC only.

### RNA-Seq *De Novo* Assembly and Transcriptome Assessment

The obtained clean reads of all 12 samples were assembled using Trinity v2.4.0 with the paired-end model and default K-mer value of 25. The *de novo* assembly was merged and clustered using CDHIT v4.0 to get non-redundant sequences. Furthermore, these non-redundant sequences were made transcripts using the trinity in-built script. The clean reads of each sample were mapped back to the *de novo* assembled genome through BWA-mem software with default parameters. The BAM files were handled by samtools. The number of reads mapped to genes was calculated using samtools v0.1.19. The expression difference of each transcript between different samples was calculated using DESeq2 R package. False discovery rate (FDR) values less than 0.01 and |log2 (fold change)| ≥2 were considered significant differences at the expression level. The transcript abundance was normalized by the fragments per kilobase of transcript per million mapped reads (FPKM) value.

### Gene Functional Analysis

To annotate the assembled transcripts, sequences were aligned by BLASTX (*e*-value <1e^−5^) to protein databases, including the non-redundant protein (NR) database, Swiss-Prot, and Kyoto Encyclopedia of Genes and Genomes (KEGG) pathway database. A GO enrichment analysis was conducted for the transcripts according to biological process, cellular component, and molecular function ontologies using Blast2GO software (Liu et al., 2013; Calzadilla et al., 2016). The GO annotation functional classifications were determined using WEGO software for the distribution of gene functions (Ye et al., 2006). GO functional enrichment and KEGG pathway enrichment analysis were also tested at a significance cutoff of *p*-value. All the *p*-values were adjusted with the criterion of Bonferroni correction. We selected the corrected *p*-value of 0.05 as the threshold to determine significant enrichment terms of the gene sets. The MapMan analysis was also conducted to provide a graphical overview of the metabolic and regulatory pathways for the detected genes using the MapMan tool, and the mapping file of ricebean for all the samples was generated using the Mercator tool.

### Candidate Gene Identification and Their Domain Analysis

The candidate genes for seed development-related traits were identified on the basis of similarity (BLASTX with similarity >80% and *e*-value <0.001) with genes responsible for similar traits in other species, including *Arabidopsis*, *Phaseolus vulgaris*, and *Vigna* species (*V. radiata* and *V. angularis*). Furthermore, the candidate genes were also validated *in silico* on the basis of their domain analysis. The amino acid sequences of the identified candidate genes were predicted and compared against the Pfam protein database using HMMER 3.0 (*e*-value ≤ 1e^−10^) to obtain candidate gene domain/family annotation information. A heatmap was also generated for the candidate genes on the basis of their expression in both the genotypes at different times of development. The heatmap was made using an in-house R script.

### Simple Sequence Repeats Identification and Primer Design

The MIcroSAtellite (MISA) search engine (http://pgrc.ipk-gatersleben.de/misa) was employed for the identification of SSRs. The minimum numbers of repeats used for selecting the SSRs were ten for mononucleotide-based loci, six for dinucleotide loci, five for trinucleotide loci, and three for all larger repeat types (tetra- to hexanucleotide motifs). For validation, 50 SSR motifs were randomly selected, that is, 25 for dinucleotide and trinucleotide each. The primers for these selected SSR motifs were designed based on flanking sequences using Primer3 software (http://sourceforge.net/projects/primer3) with targeted size of PCR products in the range between 100 and 300 bp, primer length between 18 and 22 bp, GC content between 40 and 70, and melting temperature of 50–60°C.

### Simple Sequence Repeats Validation

DNA was isolated from young leaves of eight accessions of *Vigna* species including *V. umbellata* (6), *V. mungo* (1), and *V. radiata* (1) by following the protocol described in the DNeasy plant mini kit (QIAGEN, Hilden, Germany). DNA concentration was measured using a NanoDrop™ 2000 spectrophotometer (Thermo, United States), and DNA quality was analyzed using 0.8% agarose gel. A working stock of DNA was (10 ng/µl) prepared with nuclease-free water for polymerase chain reaction (PCR) for SSR amplification.

For the SSR amplification, 20 µl reaction mixture containing 4 µl genomic DNA (40 ng), 10 µl Taq Polymerase 2X Master Mix (United States), 0.8 µl primers (10 pM), and 5.2 µl nuclease-free water were used. For the amplification, the following thermal conditions were carried out: initial denaturation of 94°C for 3 min, then 35 cycles of 94°C for 30 s, primer annealing at 55°C for 45 s, extension at 72°C for 1 min, and final extension for 10 min. PCR products were separated using high-resolution metaphor agarose gel (3%) electrophoresis. Furthermore, the dendrogram of the genotypes was generated using the hierarchical clustering algorithm in DARwin v6.0.21 software (https://darwin.cirad.fr/).

## Results and Discussion

### Transcriptome Sequencing and *De Novo* Assembly

To obtain a comprehensive transcriptome profile of ricebean 12 RNA libraries were sequenced, and a total of 94.35 Gb raw data were generated. For these 12 samples, approximately, 98.50–99.80% of reads passed the quality control, and 98.60–99.60% of the clean reads were mapped back to the *de novo* assembled ricebean genome. On average, raw data of the seed transcriptome at 5 DPA and 10 DPA had 50.33 and 48.66% GC content, respectively, while after trimming, the GC content of clean data at 5 DPA and 10 DPA was 48.66 and 49.33%, respectively, which is similar to the GC content reported in the previous study of ricebean (Chen et al., 2016; Table 1).

**TABLE 1:** Summary of RNA-Seq data for 12 samples of ricebean at 5 DPA and 10 DPA.

Genotype	Replicate	Time point	Read before quality control	Read after quality control	GC%
Bold (IC426787)	Replicate 1	5 DPA	28,178,488	28,081,924	49
		10 DPA	32,397,064	32,223,234	52
	Replicate 2	5 DPA	20,042,440	19,897,123	51
		10 DPA	23,598,201	23,252,956	48
	Replicate 3	5 DPA	32,043,486	31,869,247	51
		10 DPA	26,455,283	26,137,490	46
Small (IC552985)	Replicate 1	5 DPA	27,160,408	27,037,555	49
		10 DPA	21,272,208	21,053,611	50
	Replicate 2	5 DPA	24,589,394	24,425,550	49
		10 DPA	25,209,773	25,053,008	49
	Replicate 3	5 DPA	26,969,114	26,850,966	48
		10 DPA	24,511,033	24,368,871	49

The obtained clean reads of all 12 samples were assembled using Trinity (v2.4.0) with default parameters. The assembled transcriptome consists of 218,486 super transcripts with an N50 value of 1,041. The number of transcripts generated in the current study is comparable to previous studies. In terms of N50, the ricebean had a higher N50 value than field pea (781) and chickpea (441) (Pradhan et al., 2014; Sudheesh et al., 2015) and less value than mungbean, common bean, and adzuki bean (Hiz et al., 2014; Chen et al., 2015b; Chen et al., 2016). These results indicate the good quality of ricebean transcriptome.

**FIGURE 2:**
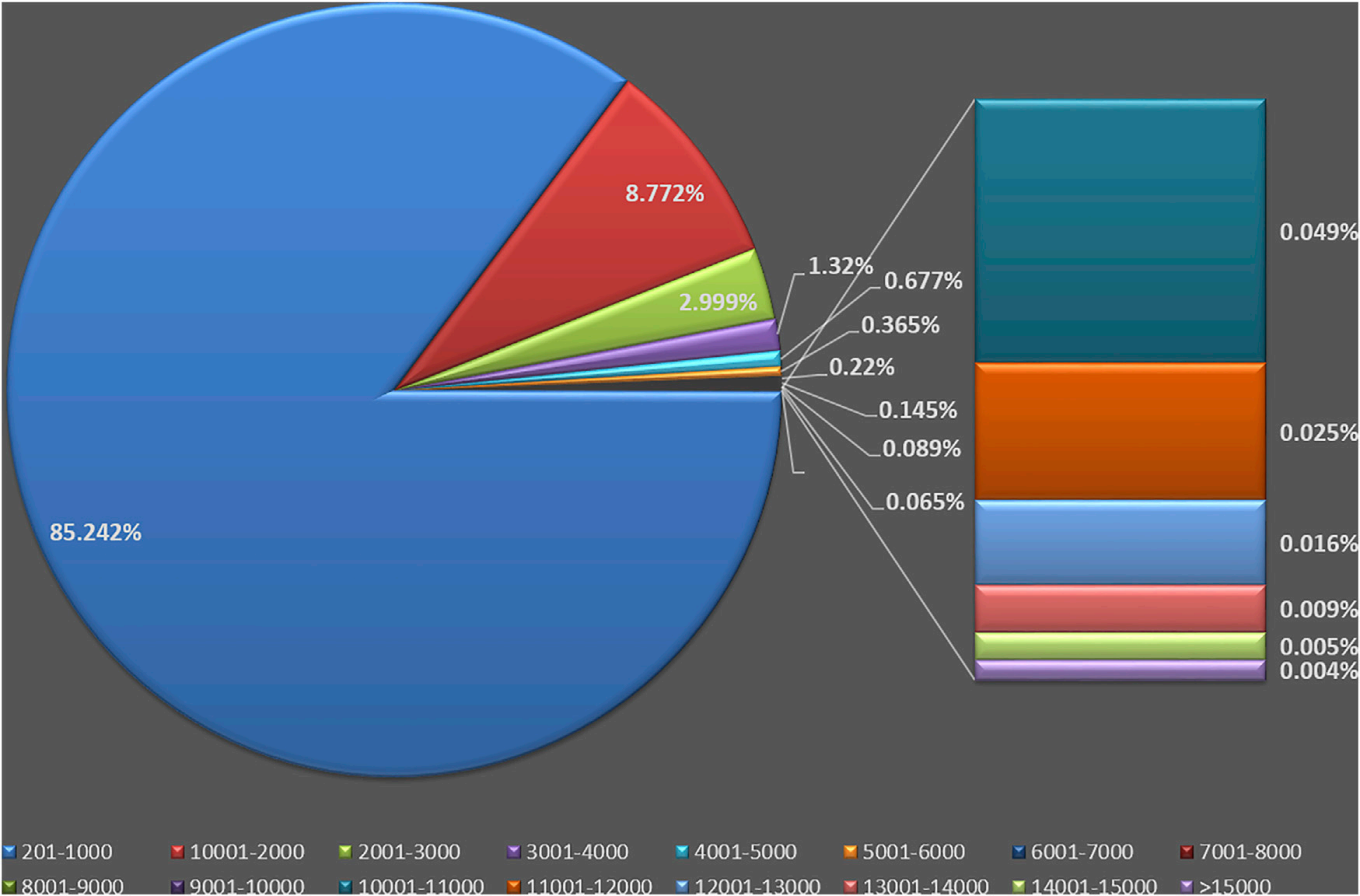
Sequence length distribution of the assembled transcripts.

The lengths of the transcripts ranged from 201 to 15,828 bp, with an average length of 669 bp, which is less than other *Vigna* species like cowpea (871 bp) and mungbean (874 bp) but more than that of black gram (443 bp) (Chen et al., 2015b, 2017; Souframanien and Reddy, 2015). Of these transcripts, 146,622 (67.11%) were 201–500 bp; 39,620 (18.13%) were 501–1,000 bp; 12,654 (5.79%) were 1,001–1,500 bp; 6,511 (2.98%) were 1,501–2,000 bp; 3,986 (1.82%) were 2,001–2,500 bp; 2,567 (1.17%) were 2,501–3,000 bp; and 6,526 (2.99%) were more than 3,000 bp in length (Figure 2). The developed assembly showed ∼100% back mapping of total and important reads, and this shows that our assembly had vast and proper mapping quality for the generated reads. The high percentage of reads mapping back to the *de novo* assembled transcriptome is a quality metric that provides an assessment of assembly entirety (Hornett and Wheat, 2012).

### Differential Expression Analysis

In this study, a comprehensive transcriptome analysis has been performed with the aim to reveal those gene expression changes that, independently of the genotype diversity, are involved in controlling seed size in ricebean. Comparative transcriptome analysis was performed between two genotypes with contrasting seed size at two time points, namely, 5 and 10 DPA. A similar type of study using two genotypes with a contrasting seed size has also been done in the peanut (Li et al., 2019b). The expression profile was checked for the individual genotypes across the time points (B5_B10, S5_S10) as well as between the genotypes at each time point (B5_S5, B10_S10) (Supplementary Table S1). False discovery rate (FDR) values less than 0.01 and |log2 (fold change) |≥2 were considered significant differences at the expression level.

**FIGURE 3:**
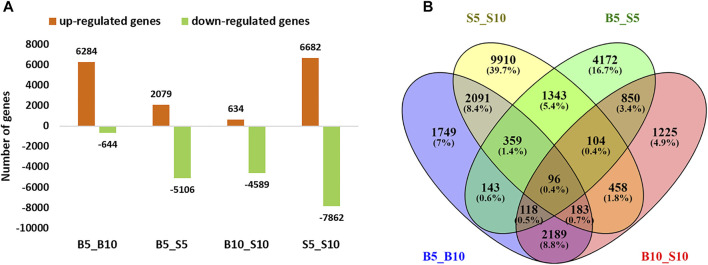
Differentially expressed genes in the two genotypes of ricebean at two time points, i.e., 5 and 10 DPA. **(A)** Comparison of DEGs representing the share of overlapped and non-overlapped transcripts in bold and small genotypes at 5 and 10 DPA. **(B)** Number of upregulated and downregulated significant genes in bold and small genotypes.

While evaluating the expression difference individually for the bold genotype across the time point (B5_B10), 6,928 differentially expressed genes were identified. In B5_B10, the number of upregulated genes (6,284) were higher than downregulated genes (644), suggesting that these upregulated DEGs might be responsible for the increase in seed size. Similarly, a small genotype across the time point (S5_S10) contributed to 14,544 DEGs (Figure3A; Table 2). In contrast to B5_B10 expression results, S5_S10 had a high number of downregulated genes (7,862) in comparison with the upregulated genes (6,682), indicating that these downregulated genes might be repressing any transcriptional activity or downstream pathways resulting in the small size of ricebean seeds (Li et al., 2019b). To gain a better understanding of molecular processes/regulatory networks associated with the seed size in ricebeans, the pattern of differentially expressed genes was analyzed between genotypes in each time point and across the time point using a Venn diagram (Figure 3B).

**TABLE 2:** Summary of significant DEGs identified in ricebean.

Comparison	Total DEG	Total significant DEG	Significantly upregulated DEG	Significantly downregulated DEG
B5_B10 DPA	276,372	6,928	6,284	644
B5_S5 DPA	227,479	7,185	2,079	5,106
B10_S10 DPA	264,964	5,223	634	4,589
S5_S10 DPA	220,089	14,544	6,682	7,862

We also identified common genes between the individual genotype across time points (i.e., B5_B10 and S5_S10) as well as between the genotypes at each time point (B5_S5 and B10_S10). In case of B5_B10 and S5_S10, in total, 2091 DEGs were common. On the other hand, 850 DEGs were common between B5_S5 and B10_S10 (Supplementary Table S2). The comparative gene expression analysis indicated that a relatively large amount of the transcriptional program operating during seed development or maturation is shared between both the genotypes. The same results have been observed in the case of common bean, where 2,487 DEGs were shared by two contrasting genotypes (González et al., 2021).

### Gene Ontology Analysis of the Transcriptome

To infer the biological processes and the functions related to seed development stages, gene ontology analysis was conducted for differentially expressed genes in terms of their biological involvement, target cellular component, and molecular function using Blast2GO. Out of total 33,880 DEGs, 16,002 DEGs contributed to GO terms. In core GO annotation, 7,002 (25.37%) genes annotated for biological process (BP), 12,069 (43.72%) for molecular function (MF), and 8,533 (30.91%) for cellular components (CC). The highest number of GO terms were observed in the case of S5_S10 (44.10%), followed by B5_B10 (23.89%), B10_S10 (16.91%), and B5_S5 (15.09%).

In case of the bold genotype across the time point (B5_B10), out of 6,928 DEGs, only 3,764 were annotated, constituting 1,696, 2,046, and 2,864 GO terms for BP, CC, and MF, respectively. However, in S5_S10, we observed 7,158 annotated DEGs from 14,544 DEGs and 2,954, 3,909, and 5,312 GO terms for BP, CC, and MF, respectively. On the other hand, in the case of between the genotype at the first time point (B5_S5), 2,537 DEGs were found to be annotated as compared B10_S10, in which 7,158 DEGs were annotated. In case of BP, 1,075 and 2,954 GO terms were identified in B5_S5 and S5_S10. Similarly, 2,046 and 3,909 GO terms were found for the cellular component function in B5_S5 and S5_S10, respectively, whereas in the case of molecular function, B5_S5 and S5_S10 consisted of 1,875 and 5,312 GO terms, respectively (Supplementary Table S3).

We have also illustrated the top or enriched functions in terms of BP, MF, and CC for both the genotypes. For example, the top biological activities include “cellular process,” “nitrogen compound metabolic process,” “small molecule metabolic process,” “cellular component organization,” “regulation of metabolic process,” “response to stress,” “cell wall organization,” cellular response to stimulus,” and developmental process. All these results indicated the biological process of DEGs vary over a broad range of terms. These enriched GO terms for BP indicate that hormone and environment stimuli played a vital role in ricebean seed/pod development. A similar type of results was also found in peanut pod development (Zhu et al., 2014).

**FIGURE 4:**
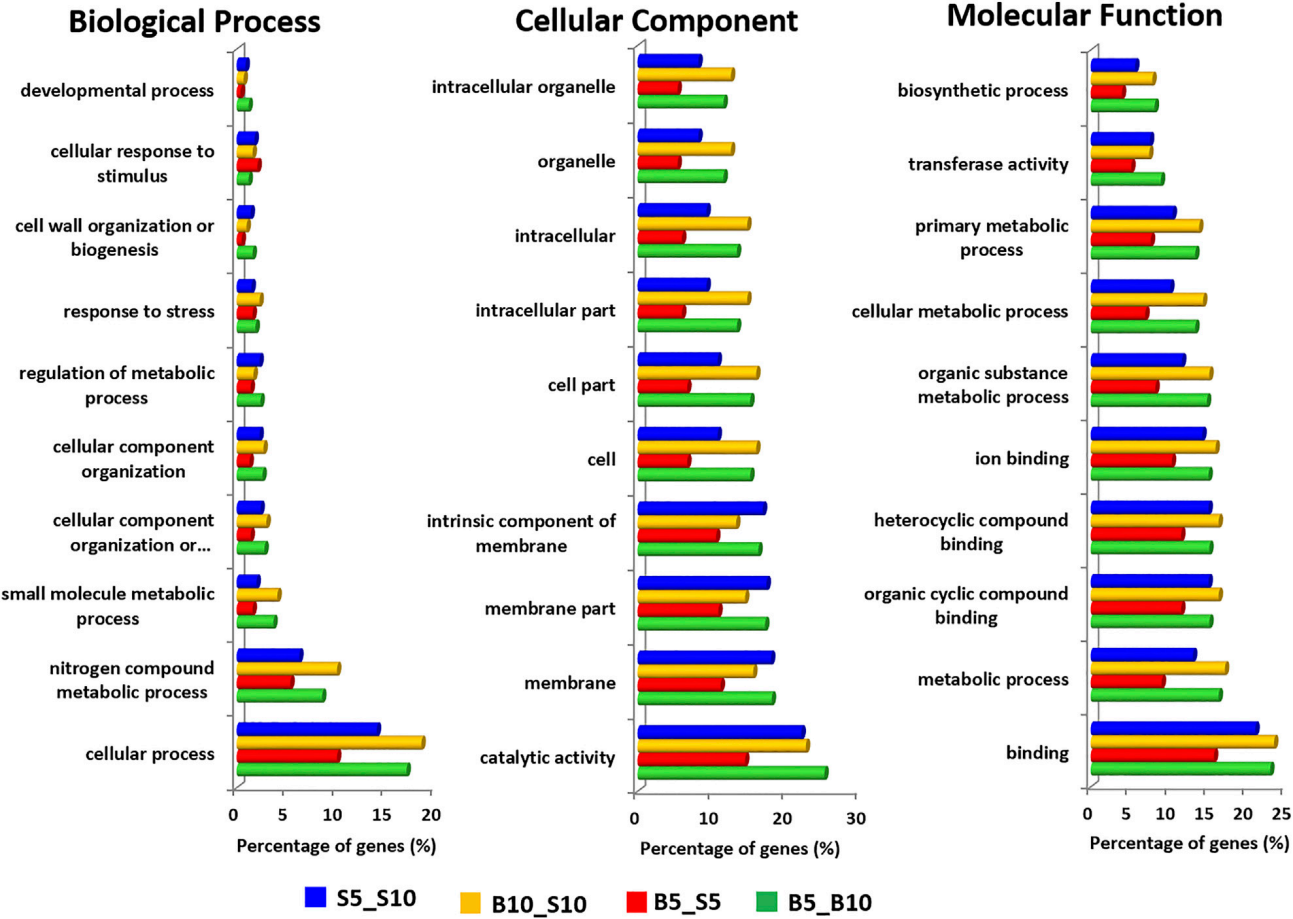
Gene ontology (GO) annotation of differentially expressed genes in ricebean summarized in three main categories: biological process, cellular component, and molecular function.

Similarly, in the case of MF, bold and small genotypes were identified to be involved in “binding,” “metabolic processes,” “organic cyclic compound binding,” “heterocyclic compound binding,” “ion binding,” “transferase activity,” and “biosynthetic processes.” However, on the other hand, cellular component activities include “catalytic activity,” “membrane,” “membrane part,” “intrinsic component of the brain,” and “intracellular” and cellular activities” (Figure 4). Similar results for MF and CC were observed in the pod development of peanuts (Zhu et al., 2014).

### MapMan Analysis

For comprehensive assessment of gene expression network dynamics in a developing seed of bold and small genotypes, identified DEGs were mapped onto metabolic maps using the MapMan tool and categorized into BINS on the basis of their functional groups. We could observe various functional groups of genes activated at different stages of seed development. Interestingly, we noticed a major variation between the bold and small genotypes with respect to genes related to important functions like those involved in different aspects of metabolism and signaling or regulation. A detailed analysis of genes expressed in these categories that actually distinguish the two genotypes was considered relevant, and a major emphasis was therefore given to the BINS in which the genotypes were found to be involved. This analysis allowed exploration of the global activation of specific metabolic pathways and gene regulatory networks activated during ricebean seed development.

**FIGURE 5:**
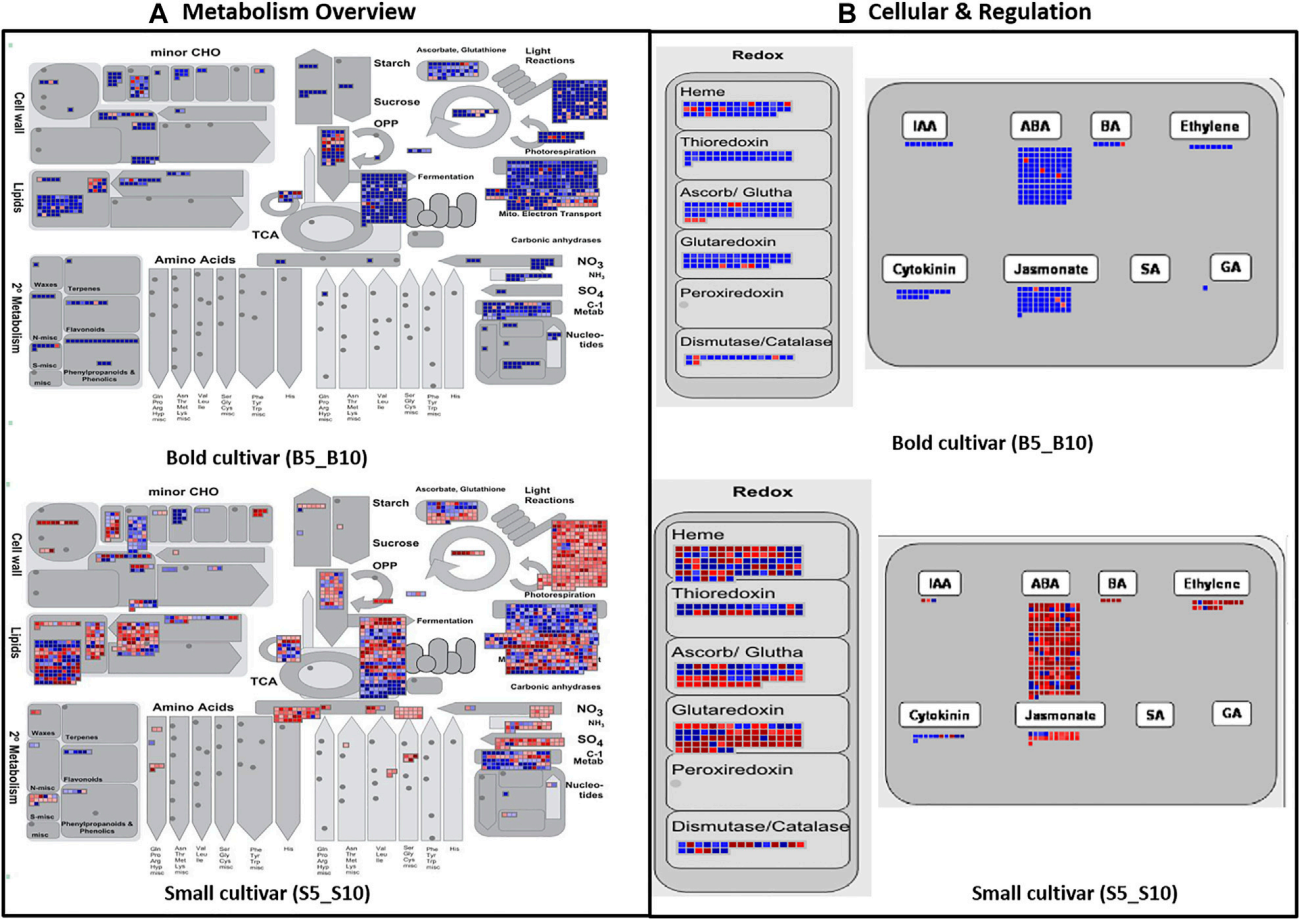
MapMan pathway representing the differential expression of genes across the time point involved in **(A)** metabolism **(B)** cellular and regulation pathway in bold and small genotypes.

**FIGURE 6:**
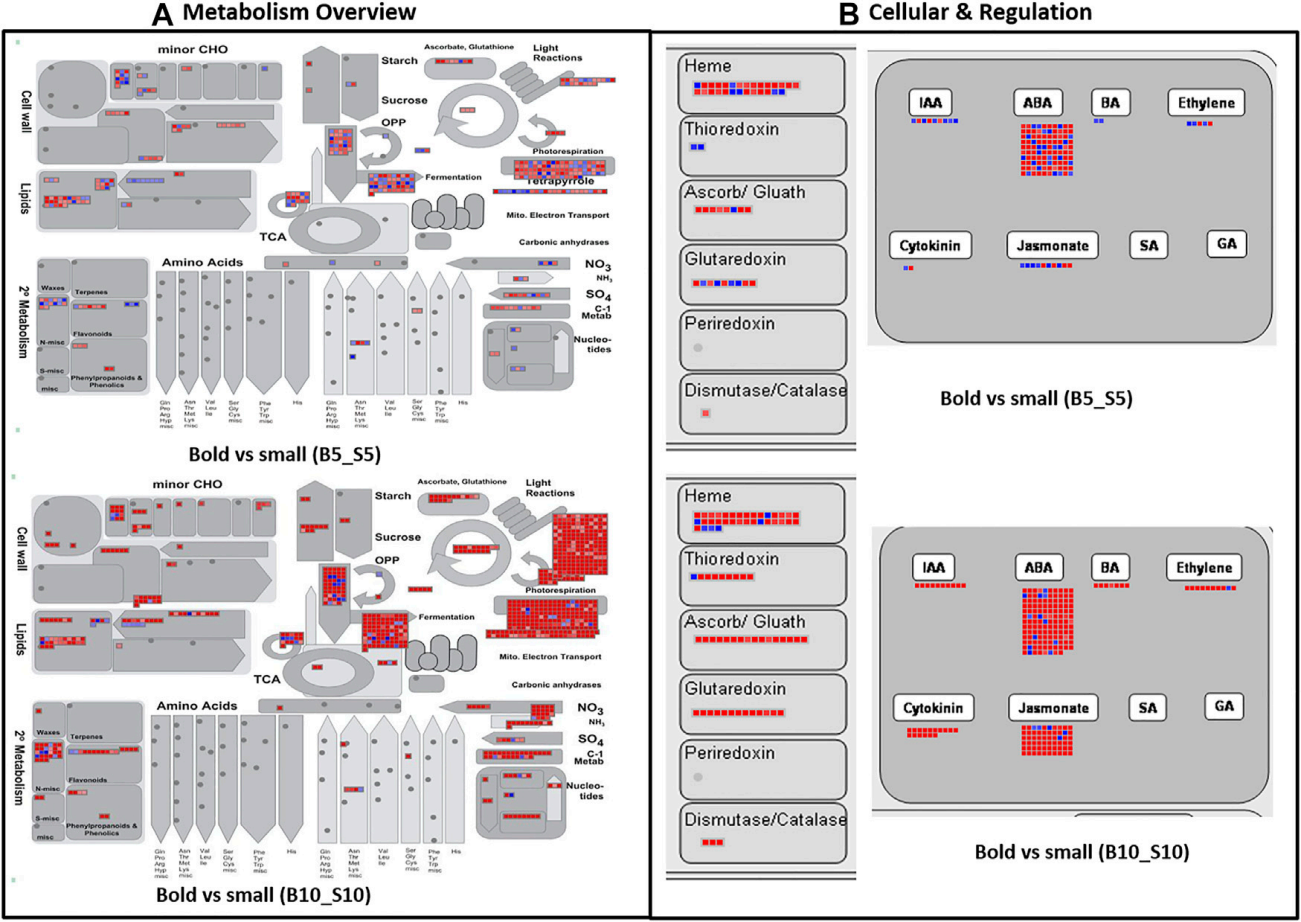
MapMan pathway representing the differential expression of genes across the genotype involved in **(A)** metabolism **(B)** cellular and regulation pathway in bold and small genotypes.

For the whole ricebean transcriptome, we annotated 13,759 transcripts with MapMan BINS of known function after running the Mercator web tool. In total, these transcripts were classified into 29 BINS. The transcripts were expressed mainly in the following categories: carbohydrate metabolism (major and minor CHO metabolism), amino acid turnover, photosynthesis, secondary metabolism, and cell wall organization (Supplementary Table S4). In the former categories, most of the transcripts were highly expressed in B5_B10, while downregulated in the case of S5_S10 (Figure 5A). B5_B10 and S5_S10 shared 17 pathways, but only two pathways were found in B5_B10 such as RNA processing and polyamine metabolism, indicating that these two pathways triggered after 5 DPA. Similarly, while comparing expressed transcripts between the genotype at the same time points (i.e., B5_S5 and B10_S10), 18 categories were the same, except the polyamine metabolism which was detected only at the second time point, that is, 10 DPA, which also confirms our previous result that polyamines activate only in the case of bold genotype after 5 DPA of seed development (Figure 6A).

In our study, photosynthesis-related genes were highly enriched in bold genotypes in comparison with the small genotype which is in similarity with the previously published reports (Zhu et al., 2014; Clevenger et al., 2016; Sinha et al., 2020). The main role of photosynthesis in seed development is reported to increase the internal oxygen content and to control biosynthetic fluxes by improving the energy supply (Borisjuk et al., 2004), and it can also affect the metabolism in a number of distinct ways (Ruuska et al., 2004). Our results indicate that many metabolic genes are most active during ricebean seed filling, which aligns with previous studies on *M. truncatula* and *P. sativa* where approximately half of the seed-regulated genes were assigned to metabolic pathways (Benedito et al., 2008; Liu et al., 2015).

Furthermore, some DEGs are also mapped to hormone metabolic pathways. Majority of the genes associated with biosynthesis and response of many phytohormones like IAA, ABA, BAP, ethylene, cytokinin, jasmonate, and gibberellic acid were upregulated in the case of bold genotypes (B5_B10) as compared to small genotypes (S5_S10), in which most of the genes were downregulated (Supplementary Table S5; Figure 5B), whereas in case of B5_S5 and B10_S10, mixed expression of phytohormones was observed (Figure 6B); a complex regulatory network triggers the initiation of seed development, maturation, and accumulation of storage products. Several studies suggested the vital role of phytohormones in pod and seed development (Zhu et al., 2014; Huang et al., 2017; Wan et al., 2017; Kumar et al., 2019; Sinha et al., 2020). In 2017, a study demonstrated the role of phytohormones in various aspects of plant hormone homeostasis including biosynthesis, metabolism, receptor, and signal transduction (Xu and Huang, 2017).

### Kyoto Encyclopedia of Genes and Genomes Pathway Analysis

**FIGURE 7:**
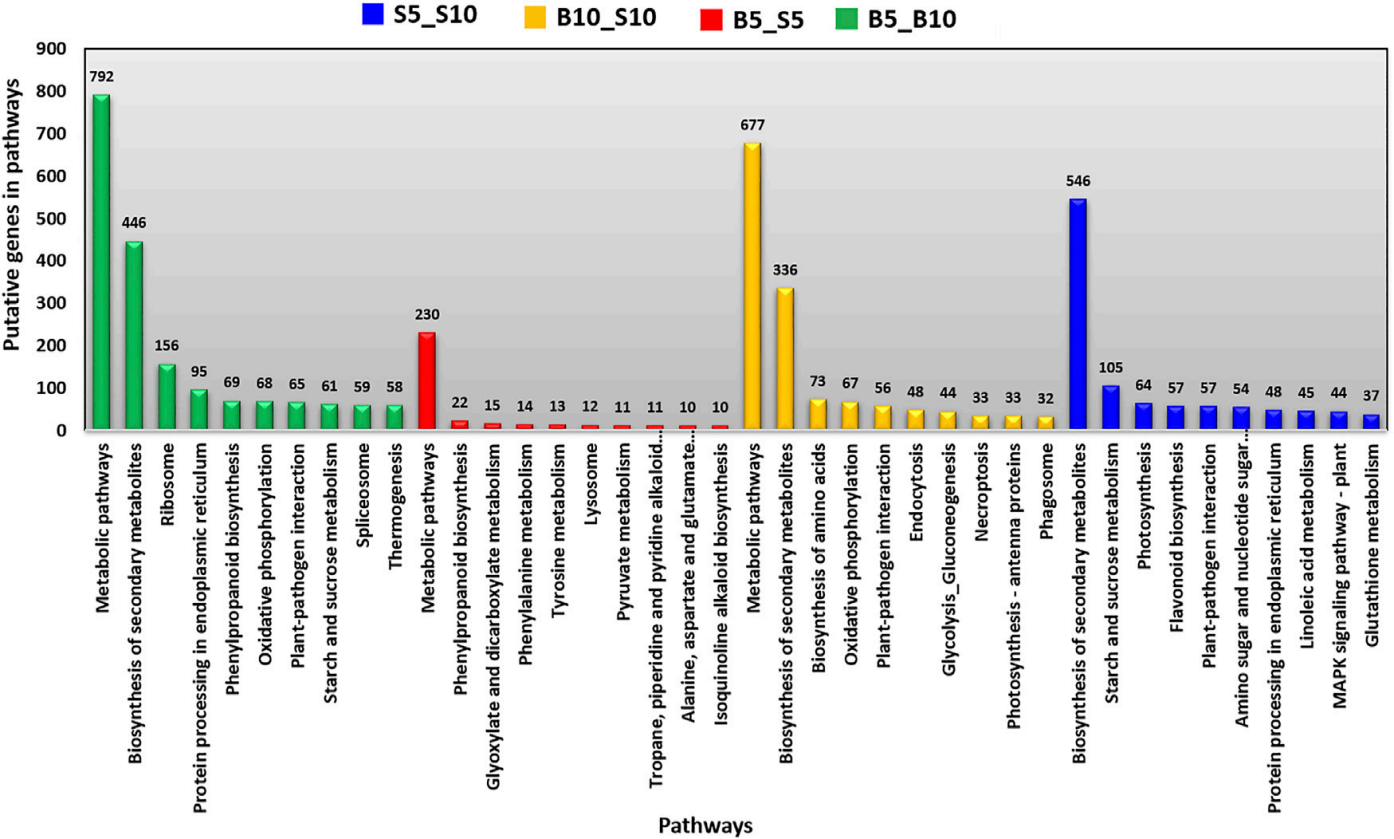
List of top 10 pathways revealed by KEGG enrichment analysis.

The KEGG pathway enrichment analysis was conducted for two contrasting genotypes at both time points (i.e., 5 DPA and 10 DPA) at a *p*-value <0.05 using the KEGG database server. The KEGG pathway enrichment analysis indicated that 7,178 transcripts obtained hits in the KEGG database, and those transcripts were associated with 106 unique pathways. The 7,178 transcripts included 3,112, 434, 2,103, and 1,529 transcripts with respect to B5_B10, B5_S5, B10_S10, and S5_S10, respectively. The pathway enrichment analysis of DEG conducted between different combinations, B5_B10, B5_S5, B10_S10, and S5_S10, revealed involvement the of 7, 52, 458, and 35 pathways, respectively. In case of B5_B10 and S5_S10, from the top 10 pathways, four pathways, namely, biosynthesis of secondary metabolites, protein processing in the endoplasmic reticulum, plant–pathogen interaction, and starch and sucrose metabolism were common. On the other hand, between the genotypes at both the time points (i.e., B5_S5 and B10_S10), only one pathway i.e., metabolic pathway—was common. The top 10 pathways among the time points for both genotypes as well as between the genotypes at both the time points are represented in Figure 7.

In KEGG pathway–based analysis, we observed a clear difference in the expression of some phytohormones which regulates seed development, including auxin, cytokinin, gibberellin, and ethylene. The differential expression of these phytohormones was also observed in our MapMan analysis. This was not surprising since phytohormones control or influence all aspects of plant growth and reproduction, including seed germination, growth of roots, stems and leaves, plant flowering, seed development, seed fill, and seed dormancy. The expression pattern of key genes involved in biosynthesis and signaling of important phytohormones was compared between small and bold seeded genotypes for their possible role in determining seed size.

### Auxin Pathway

**FIGURE 8:**
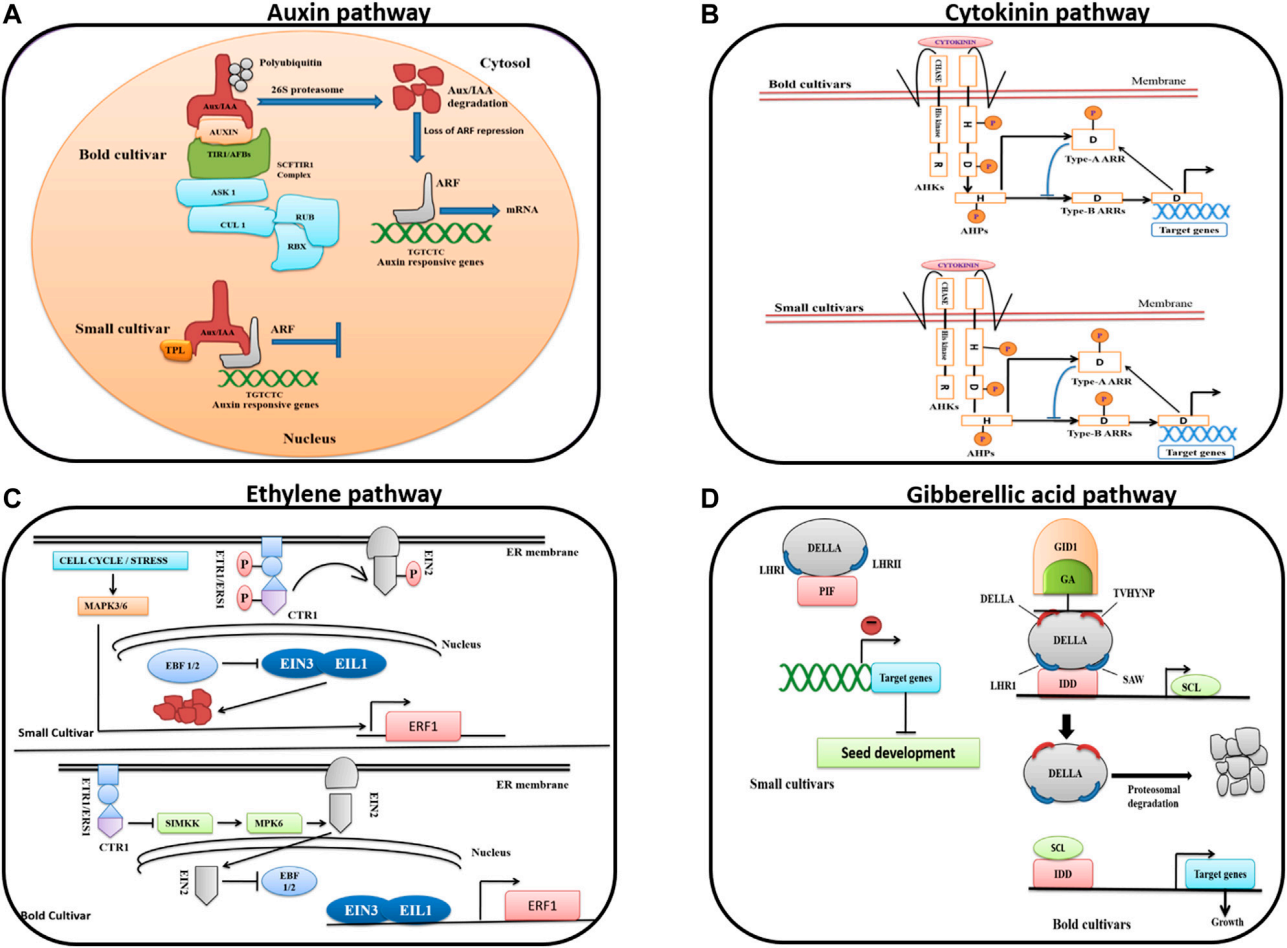
Phytohormone pathways important for seed development are represented in two contrasting genotypes of ricebeans on the basis of their expression and involvement in the enriched KEGG pathways. **(A)** Auxin signaling pathway. **(B)** Cytokinin pathway. **(C)** Ethylene pathway. **(D)** Gibberellic acid pathway.

Auxin regulates many aspects of plant growth and development, including embryogenesis (Möller and Weijers, 2009), the architecture of the root system (Benková et al., 2003), gravitropism (Rashotte et al., 2003), phototropism (Blakeslee et al., 2004), initiation and radial positioning of plant lateral organs, and cell elongation (Reinhardt et al., 2000; Christian et al., 2006). Auxin is sensed by its receptor protein such as *TRANSPORT INHIBITOR RESPONSE 1/AUXIN-SIGNALING F-BOX* proteins (*TIR1/AFBs*) which mediate the auxin signaling pathway and centered on a ubiquitin-dependent *Skp1-Cullin-F-box* (*SCF*)^*TIR1/AFBs*^ protein complex to regulate the *Aux/IAAs*-*ARFs* flow (Leyser, 2003; Figure 8A). The TIR receptor protein confers substrate specificity and target-specific *Aux/IAA* proteins for degradation via the *SCF* ^*TIR1/AFBs*^ protein complex, in the presence of auxin. The degradation of *Aux/IAA* leads to switching on transcriptional expression of a range of genes including auxin responsive factors (*ARFs*) which in turn regulate the expression of several other genes that have a role in auxin-mediated plant growth and development.

The KEGG pathway expression–based analysis revealed a clear difference in the auxin signaling pathway in two contrasting ricebean genotypes, which is also in accordance with our MapMan results where auxin signaling related genes showed higher expression in the bold genotype than the small genotype. We found approximately 51 genes encoding *SCF* ^*TIR1/AFB*^, *Aux/IAA*, *ARFs*, *E3* ubiquitin transferase enzyme, and *26S* proteasome, showing distinct expression dynamics in bold (B5_B10) and small (S5_S10) genotypes (Supplementary Table S6). The three key signaling elements *TIR1/AFBs*, *Aux/IAAs*, and *ARFs* have also been identified in different species including *Arabidopsis* (Chapman and Estelle, 2009), populus (Kalluri et al., 2007), and rice (Jain et al., 2005; Parry et al., 2009; Shen et al., 2010)*.* Similarly, several studies focused on the role of *AUX/IAA* in determining the seed size with the influence of the expression of a gene in *AUX* biosynthesis (*ZmTar3*, *ZmTar1*, and *ZmYuc1*) and signaling (auxin efflux carriers, *PIN*, and *ARF2*) (Schruff et al., 2006; Bernardi et al., 2016). Homologs of *ZmYuc1*, *PIN*, and *ARF2* were significantly differentially expressed during tartary buckwheat seed development (Huang et al., 2017). The high expression of DEGs in bold genotypes corresponds to cell division, and expansion is faster to form larger size seeds at these stages.

The upregulation of *SCF* ^*TIR1*^, *E3* ubiquitin transferase enzyme, and *26S* proteasome was found in the bold genotypes, indicating the degradation of *Aux/IAA* and release of *ARFs* to modulate the expression of their target genes including *SMALL AUXIN UP RNA* (*SAUR*), Gretchen Hagen 3 (*GH3*), and indole-3-acetic acid–inducible gene (*Aux/IAA*), while in case of small genotypes, *SCF* ^*TIR1*^ was not expressed, but *TOPLESS* (*TPL*) gene was upregulated, suggesting that Aux/IAA might have formed the complex with *ARFs* to block the transcriptional activity (Figure 8A; Hayashi, 2012).The induction of auxin-inducible acyl amidosynthetases, *GH3*, by the *ARF* family is the early event of auxin signaling cascade (Zhang et al., 2016). The expression of *GH3* gene was upregulated in the case of bold genotypes, while it was downregulated in the small genotypes. *SAUR* expression was upregulated in small genotypes, while downregulated in bold genotypes. The aforementioned results clearly inferred that the differential regulation of the auxin signaling pathway in bold and small genotypes might be the main factor contributing to the variation in ricebean seed size (Bai et al., 2019).

### Cytokinin Pathway

Similar to auxin, cytokinin is another important plant hormone regulating many aspects of plant growth (Tarkowski et al., 2006; Werner et al., 2008). In plants, the regulation of cytokinin is facilitated by the two-component system (TCS) which consists of four groups of proteins: histidine kinases (*AHKs*; *AHK2*, *AHK3*, and *AHK4*/*WOL1*/*CRE1*), histidine-containing phosphotransfer proteins (*AHPs*; *AHP1*-*AHP5*), type-B response regulators (type-B *ARRs*; *ARR1*, *ARR2*, *ARR10*-*ARR14*, and *ARR18*-*ARR21*), and type-A ARRs (*ARR3*-*ARR9* and *ARR15*-*ARR17*). In *Arabidopsis*, *AHK2*, *AHK3*, and *CRE1* were found to be involved in seed size (Riefler et al., 2006; Heyl et al., 2012).

Cytokinins have been reported to function in seed development, such as seed size, seed yield, embryonic growth, with the involvement of genes encoding *isopentenyl transferase* (*IPT*), *cytokinin oxidase/dehydrogenase* (*CKX*), and *histidine kinase* (*HK*) (Bartrina et al., 2011). In our study, we have also found the expression of genes such as *IPT*, *CKX*, and *HK*. *IPT* upregulation was observed only in the case of small genotypes, whereas *CKX* was upregulated in bold genotypes, and mixed expression of *HK* was noticed in both the genotypes (Figure 8B; Supplementary Table S7). The upregulation of *CKX* in bold seed genotypes hints at its possible role in determining the seed size. The *CKX* proteins are widely distributed in plants and implicated in various plant growth and developmental processes by maintaining the endogenous cytokinin level via irreversible degradation. In plant tissues, the expression of the *CKX* genes is primarily regulated by the endogenous cytokinin level. Various past studies have shown the role of *CKX* genes in the regulation of the seed size and grain yield in different plant species*.* In *Arabidopsis*, a *CKX* family gene–encoded enzyme *CYTOKININ OXIDASE 2* (*CKX 2*) has been demonstrated to be associated with large seed size *via* catalyzing irreversible degradation of cytokinin. Similarly, in rice, a Gn1a locus encoding for *cytokinin oxidase/dehydrogenase* (*OsCKX2*) is shown to be responsible for high grain yield (Ashikari et al., 2005). On the other hand, the expression of type-A *Arabidopsis* response regulator (type-A *ARRs*) genes that negatively regulates the cytokinin signaling was majorly detected in small genotypes. This suggested that type-A *ARR* genes may be repressing the cytokinin signaling pathway (Heyl and Schmülling, 2003; Lohar et al., 2004; Desbrosses and Stougaard, 2011). The inhibition of the cytokinin signaling pathway may contribute to plant and bacterial cell differentiation (Bromley et al., 2014). Mixed expression of *AHP* and type-B *ARRs* was found in both the genotypes. Phosphate transfer to type-B *ARR* proteins modulates the transcriptional changes in the nucleus and causes the expression of primary cytokinin response genes including the type-B *ARRs*.

### Ethylene Pathway

Ethylene, an “aging” hormone, has been reported to control the development of plant seeds and grains in various species (Zhong et al., 2002; Hentrich et al., 2013; Huang et al., 2013; Guo et al., 2016).Molecular evidence demonstrated ethylene’s role in the regulation of seed size and seed shape, in which genes in ethylene biosynthesis (*EIN2*, *ERS1*, and *ETR1*), signaling (*CTR1*, *ETO1*, *ETR1*, and *EIN2*), and catabolism (*ACC deaminase*) were involved (Robert et al., 2008; Walton et al., 2012). According to our results, the expression of ethylene receptors (*ERS1/2*) was higher in bold genotypes than small genotypes, whereas *CTR1*, a negative regulator of ethylene hormone showed contrasting expression with upregulation in small and downregulation in bold genotypes (Figure 8C; Supplementary Table S8). In buckwheat, the differential expression of *ERS1*, *ETO1*, *ETR1*, etc. was observed (Huang et al., 2017). In case of bold genotypes, we have noticed the high expression of *SIMKK*, *MPK6*, *EIN3*-like transcription factors, and *EIN2*, indicating positive regulation of transcriptional response in the bold genotype. In case of small genotypes, the upregulation of *ERFs* depicted that *ERF* might have shown activity after the phosphorylation via the *MPK3*/*6*-cascade, which regulates the ethylene biosynthesis, and the expression of EIN3/EIL1 was not found which possibly indicates its degradation by ubiquitination. In our samples, we found a full cascade of gene expressions in bold genotypes, while in small genotypes, the expression of genes detour from the normal expression and opted a new route for the ethylene-inducible gene expression.

### Gibberellin Pathway

Gibberellins (*GAs*) are well-known plant hormones that are widely involved in the growth and development processes. *GAs*, auxin, *ABA*, and ethylene have been involved in the regulation of seed development and pod maturation (Ziv and Kahana, 1988; Shlamovitz et al., 1995; Ozga et al., 2003). In case of bold genotypes, the expression of *GA*, *DELLAs*, and *SCF*-complex protein is upregulated, which indicates *DELLA* proteolysis; simultaneously, the upregulation of protein indeterminate domains (*IDDs*) and *scarecrow-like* proteins (*SCLs*) were also observed, which supports the feedback loop mechanism which regulate the *GA* signaling (Figure 8D). According to the feedback loop mechanism, *DELLA* initiates the expression of downstream genes, including *SCLs* by *IDD*-mediated interaction with their promoters. The subsequent increased concentration of *SCLs* enhances the *SCL3/IDD* complex synthesis while decreasing the formation of the *DELLA/IDD* complex and consequent suppression of the expression of *SCLs*, which mediates the homeostatic regulation of the downstream genes, including positive regulation of *SCLs* and *GA* signaling. In case of small genotypes, the expression of *SCF complex* protein was absent, while the expression of *DELLAs* was unregulated. Consecutively, we observed the *SCL* protein script, while *IDD* protein was completely absent. The expression of the phytochrome-interacting factor (*PIF*) protein was found, which indicates the *DELLA*-mediated inhibition of hypocotyls elongation (Supplementary Table S9).

Previous studies have revealed that genes encoding *GA2 oxidase* and *GA3 oxidase* in the GA biosynthesis pathway can affect seed development, starch biosynthesis, embryo, and seed coat development (Nakayama et al., 2002; Singh et al., 2002). The downregulation of *GA2-oxidase* was observed in our results similar to a study of the tartary buckwheat in which the downregulation of *GA2-oxidase* was also depicted during seed development (Huang et al., 2017).

The KEGG pathway and the MapMan analysis suggested the differential expression of phytohormone biosynthesis or response genes. According to the MapMan analysis, auxin, cytokinin, ethylene, and gibberellin showed contrasting expressions in both the genotypes (Figure 5B). Similarly, in terms of the KEGG pathway, we have observed how the signaling pathways of these phytohormones were different. The present work confirms that auxin, cytokinin, ethylene, and gibberellin are the important regulators of the seed size in ricebean. Our results are also in accordance with those of previous studies in other species (Riefler et al., 2006; Heyl et al., 2012).

### Candidate Gene Identification for Seed Development–Related Traits

**FIGURE 9:**
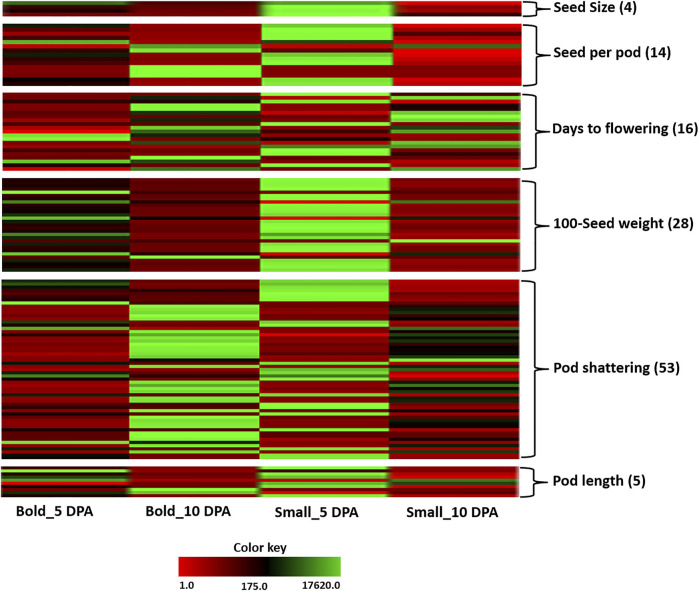
Heat map representing the differential gene expression of the identified candidate genes for six traits including seed size, 100-seed weight, seed/pod, days to flowering, pod shattering, and pod length in bold and small genotypes at 5 DPA and 10 DPA.

The expression of a number of genes starting from the anthesis to early stages of maturity may have a crucial role in determining grain size and various other pod-related traits in pulses (Pazhamala et al., 2016). In this study, candidate genes for various traits such as days of flowering, pod shattering, seed per pod, seed size, 100-seed weight, and pod length were identified from the assembled transcriptome on the basis of sequence similarity search. In total, we identified 142 genes in ricebean belonging to development-related traits on the basis of similarity search (BLASTX) and e-value. Furthermore, the candidate genes were also characterized *in silico* on the basis of their domain analysis using Pfam software. Out of 142 genes, only 120 genes showed domain similarity with their hits. Therefore, we discarded 22 genes whose domain was not matched. Hence, according to our study, we found 120 candidate genes of ricebean belonging to different development-related traits (days of flowering, pod shattering, seed per pod, seed size, 100-seed weight, and seed length) (Figure 9; Supplementary Table S10).

In terms of pod development, seed size is a key determinant for the seed or grain yield in legume crops (Amkul et al., 2020). In ricebean, we found four candidate genes for seed size encoding: *histidine kinase 2*, *delta sterol reductase*, *phosphate transporter* (*PHO1*), and *WRKY* domain–containing protein (*WRKY 40*). These genes have already been reported to be involved in seed size. For example, *Vigun05g039600* (*PHO1*) has been reported to be a positive regulator of seed development that affects both the cell size and cell number (Lo et al., 2019). Similarly, *Vigun08g217000* which codes for *histidine kinase 2* has been identified as a potential candidate gene for improved organ size during cowpea domestication (Lonardi et al., 2019), and its *Arabidopsis* ortholog *AHK2* has been shown to regulate the seed size (Riefler et al., 2006; Bartrina et al., 2017). *Vigun11g191300* encoding a *delta (24)-sterol reductase* is an ortholog of the *Arabidopsis DIMINUTO* gene which has been shown to regulate cell elongation (Takahashi et al., 1995). In foxtail millet, *Loose Panicle1*–encoded *WRKY* transcription factor regulates the seed size by increasing the length and width of the seed (Xiang et al., 2017). Hence, these genes are the strong candidates as seed size is affected by multiple pathways.

On the other hand, for 100-seed weight, 29 candidate genes were identified corresponding to *expansin*, *cytokinin dehydrogenase*, *cytochrome P450*, and response regulatory domain containing protein. The significance of these genes as candidate loci related with the 100-seed weight is supported by the work done on *Arabidopsis*, where orthologs of the candidate genes in the cytokinin pathway have been shown, in transgenic studies, to regulate seed size and/or weight (Daele et al., 2012). Our findings are also in accordance with the common bean in which type-B regulators were found to be involved in the activation of downstream genes in the cytokinin pathway, and the genes encoding cytokinin dehydrogenase regulates the pathway by degrading active cytokinin (Hwang et al., 2012; Schmutz et al., 2014). Likewise, in *Arabidopsis*, *expansins* increased grain size and also improved grain production (Bae et al., 2014). Recent studies have also associated *expansins* with grain size and weight in wheat and tomato (Muñoz and Calderini, 2015; Brinton et al., 2017). *TaCYP78A3* in wheat and *CYP78A5* in *Arabidopsis* encodes the *cytochrome P450*, which positively correlates with seed size and seed weight (Ma et al., 2015; Tian et al., 2016b).

Like other development-related traits, flowering time is also an important trait because several agronomical traits such as quality of the grain and grain yield depend on flowering time. For days to flowering trait, we identified 21 candidate genes in our dataset encoding protein *Flowering Locus T-like*, *GIGANTEA-like*, *cryptochrome*, and transcription factors such as *bHLH*, *ERF*, and *PIF-3.* Most of the candidate genes of days to flowering had high expression in case of 10 DPA, instead of 5 DPA. In rice, *florigen* is encoded by *RICE FLOWERING LOCUS T 1* (*RFT1*) and the orthologs of *Arabidopsis* FT and plays important role in heading date, influencing yield traits in rice (Tamaki et al., 2007; Komiya et al., 2009), whereas *GIGNANTEA*-like genes observed in the regulation of many genes which influence the circadian clock, blue light photoreceptor, and flowering time have also been reported in *Arabidopsis* (Hayama et al., 2003; Fornara et al., 2009). Similar to rice results, the *Flowering Locust T*-*like* in ricebean might help in the improvement of yield.

On the other hand, the number of seeds per pod might be useful for increasing the seed yield of ricebean. We identified 15 candidate genes for seeds per pod trait having annotations like *MAPK*, *NAC*, *MALE STERILE 5*, and *ABSCISIC ACID-INSENSITIVE 5*-like protein*. Vigun03g187300* (*ABA-insensitive 5-like protein 6*) is an ABA-responsive element (*ABRE*)–binding factor that regulates *ABRE*-dependent gene expression (Nakashima and Yamaguchi-Shinozaki, 2013). In *Arabidopsis*, ABA deficiency reportedly decreases the number of seeds per siliqua (Cheng et al., 2014). Hence, the higher expression of this gene in bold genotypes implies an increase in the number of seeds per pod that could result in the improvement of the ricebean yield. The *Vigun05g126900* gene, encoding *MALE STERILE 5*, was selected as a candidate gene in zombie pea (Amkul et al., 2020). In a previous study on *Arabidopsis*, mutations to *MALE STERILE 5* resulted in the development of “polyads” (i.e., tetrads with more than four pools of chromosomes following male meiosis) (Glover et al., 1998). Plants that are homozygous for the *MS5* recessive allele apparently revealed arrested growth and harvested empty siliques, whereas in plants that are heterozygous for *MS5*, siliqua elongation and seed set are less repressed (Glover et al., 1998). In case of the pod length, five candidate genes in ricebeans have been identified, mostly corresponding to the auxin response factor. *Glyma.07G134800*, an ortholog of *Arabidopsis*, was also associated with the auxin pathway (Jiang et al., 2018).

Furthermore, we have also identified a few candidate genes associated with pod shattering which is considered to be an undesirable agronomical trait. We identified maximum candidate genes (i.e., 57) for this trait in our ricebean study. Out of the 57 candidate genes, 18 genes encode transcription factors like *AP2/ERF*, *WRKY*, and *NAC*, whereas the rest of the genes were involved in cellulose synthase and serine/threonine protein kinase. The candidate genes for pod shattering have also been identified in other legumes including *Vigun02g095200* (cellulose synthase), *Vigun03g306000* (*NAC* domain transcription factor), and zombi pea (Suanum et al., 2016; Lo et al., 2018; Takahashi et al., 2019; Amkul et al., 2020; Watcharatpong et al., 2020). In *Sorghum propinquum*, *WRKY* modulates the flower and seed development and lignin deposition, and it is also found to be involved in pod shattering (Tang et al., 2013). Recently, in rice, *AP2* transcription factor–coding gene *SHATTERING ABORTION1* (*SHAT1*) was observed having a crucial role in pod shattering. Two genes encoding *NAC* in *Vigna unguiculata* were found to be involved in cell wall biosynthesis and hence influencing the pod shattering (Zhou et al., 2012; Lo et al., 2018). The identification of pod shattering genes may reduce preharvest yield damages in ricebean, resulting in a more efficient yield. Thus, pod indehiscence may be a valuable trait during seed harvesting, making it a main concern during crop domestication (Amkul et al., 2020).

To support our findings related to candidate genes, we performed a comparative analysis of the identified candidate genes with our MapMan and KEGG pathway results. Out of 120 candidate genes, 23 genes matched with the MapMan results (Table 3). For example, the expression of eight candidate genes of 100-seed weight and seed size were only shown in the small genotype encoding *PHO1*, *cytokinin dehydrogenase*, A-type cytokinin *ARR* response negative regulator, etc. Similarly, for bold genotypes, only one gene, *aB10dtrinity_dn14585_c0_g1_i2*, for a seed was upregulated, revealing a high number of pods in bold genotypes as compared to the small genotype. On the other hand, in terms of time point, three genes (*cS5dtrinity_dn10996_c2_g4_i3*: seed size; *cS5dtrinity_dn11557_c0_g1_i4*: seeds/pod; and *aB10dtrinity_dn30303_c0_g10_i1*: days to flowering) were detected only at the first time point, that is, 5 DPA. Two genes (*aB10dtrinity_dn33078_c0_g1_i1*: 100-seed weight and *bS10d1trinity_dn10624_c1_g9_i1*: pod shattering) were found to be highly expressed only in bold genotypes, whereas nine genes encoding alpha class *expansins* were found to be downregulated, specifically in the small genotype.

**TABLE 3:** List of candidate genes matched with our MapMan results.

MapMan category	Candidate ricebean gene ID	Description	B5_B10	S5_S10	B5_S5	B10_S10	Trait
Amino acid metabolism	*cs5dtrinity_dn11557_c0_g1_i4*	Histidinol-phosphate aminotransferase	—	—	2.23	—	Seeds/pod
Cell wall organization	*bs10d1trinity_dn10624_c1_g9_i1*	Catalytic component CesA of cellulose synthase complex	2.34	—	—	—	Pod shattering
	*ab10dtrinity_dn33078_c0_g1_i1*	Alpha-class *expansin*	7.38	—	—	—	Seed weight
	*ab4dtrinity_dn10598_c3_g1_i1*	Alpha-class *expansin*	—	−3.88	—	—	Seed weight
	*ab10dtrinity_dn30367_c2_g3_i1*	Alpha-class *expansin*	—	−3.22	—	—	Seed weight
	*bb4dtrinity_dn13171_c6_g7_i1*	Alpha-class *expansin*	—	−3.13	—	—	Seed weight
	*bb10dtrinity_dn16316_c5_g6_i2*	Alpha-class *expansin*	—	−2.76	—	—	Seed weight
	*bs5dtrinity_dn13044_c1_g3_i3*	Alpha-class *expansin*	—	−2.82	—	—	Seed weight
	*cb4dtrinity_dn14045_c9_g6_i1*	Alpha-class *expansin*	—	−3.33	—	—	Seed weight
	*cb4dtrinity_dn14247_c0_g4_i1*	Alpha-class *expansin*	—	−4.18	—	—	Seed weight
	*cs5dtrinity_dn11417_c0_g2_i1*	Alpha-class *expansin*	—	−3.43	—	—	Seed weight
	*cs5dtrinity_dn12484_c15_g1_i1*	alpha-class *expansin*	—	−3.3	—	—	Seed weight
Lipid metabolism	*cs5dtrinity_dn11621_c1_g7_i3*	Sterol delta24 reductase	—	−2.09	—	—	Seed size
	*ab10dtrinity_dn14585_c0_g1_i2*	Dihydrolipoamide acetyltransferase component E2	5.3	—	—	—	seeds/pod
Nucleotide metabolism	*ab10dtrinity_dn30303_c0_g10_i1*	Uracil phosphoribosyltransferase (UPP)	—	—	−3.05	—	days to flowering
Nutrient uptake	*cs5dtrinity_dn18119_c0_g1_i1*	Phosphate transporter (PHO1)	—	−2.93	—	—	Seed size
Phytohormone action	*cs5dtrinity_dn10996_c2_g4_i3*	Receptor protein (AHK)	—	—	2.2	—	Seed size
	*ab4dtrinity_dn16153_c0_g1_i2*	Cytokinin dehydrogenase	—	−4.66	—	—	Seed weight
	*cs5dtrinity_dn9587_c0_g1_i4*	Steroid 22-alpha-hydroxylase (DWF4)	—	−3.24	—	—	Seed weight
	*as10dtrinity_dn8390_c0_g1_i1*	A-type cytokinin ARR response negative regulator	—	3.93	—	—	Seed weight
	*cs5dtrinity_dn5774_c0_g1_i1*	Cytokinin dehydrogenase	—	−3.51	—	—	Seed weight
Protein homeostasis	*bb4dtrinity_dn32604_c0_g1_i1*	Matrixin-type metalloprotease	—	−2.66	—	—	Pod Shattering
Redox homeostasis	*ab4dtrinity_dn10550_c1_g1_i11*	GDP-D-mannose-epimerase (GME)	—	−2.78	—	—	Seeds/pod

Similarly, 16 candidate genes (auxin: 2; cytokinin: 8; ethylene: 5; GA: 1) were matched with the KEGG pathway results (Table 4). The matched genes were found to be associated with several seed development–related traits like pod length, days to flowering, 100-seed weight, seeds/pod, and pod shattering. All the genes were expressed in the small genotype, except two (*ab10dtrinity_dn29885_c1_g2_i2* and *bs10dtrinity_dn12088_c3_g6_i6*) which were expressed in bold genotypes corresponding to *MAPK*.

**TABLE 4:** List of candidate genes matched with our KEGG pathway results.

KEGG pathway	Ricebean candidate gene ID	Description	B5_B10	S5_S10	Trait
Auxin	*cb10dtrinity_dn16977_c3_g12_i1*	Auxin response factor	—	−2.09	Pod length
	*cs5dtrinity_dn10719_c0_g1_i4*	Auxin response factor	—	−2.15	Pod length
Cytokinin	*ab4dtrinity_dn16153_c0_g1_i2*	Cytokinin dehydrogenase 6–like	—	−4.66	Seed weight
	*cs5dtrinity_dn10983_c0_g1_i2*	Two-component response regulator–like APRR1 isoform X4 (CCT motif, rec)	—	−2.19	Days to flowering
	*as5dtrinity_dn1065_c0_g1_i1*	HPt domain–containing protein	—	2.17	Seed weight
	*as10dtrinity_dn8390_c0_g1_i1*	Response regulatory domain–containing protein (type A)	—	3.93	Seed weight
	*bs10d1trinity_dn9417_c0_g2_i1*	HPt domain–containing protein	—	2.26	Seed weight
	*cb10dtrinity_dn35628_c0_g1_i1*	HPt domain–containing protein	—	2.71	Seed weight
	*cs5dtrinity_dn4103_c0_g1_i1*	Cytokinin hydroxylase–like	—	−2.85	Seed weight
	*cs5dtrinity_dn5774_c0_g1_i1*	Cytokinin dehydrogenase 6–like	—	−3.51	Seed weight
Ethylene	*bb10dtrinity_dn16202_c0_g2_i1*	Ethylene-responsive transcription factor *RAP2-7–like* isoform *X2*	—	2.52	Days to flowering
	*cb4dtrinity_dn13190_c1_g1_i4*	*AP2/ERF* domain–containing protein	—	3.14	Pod Shattering
	*ab10dtrinity_dn29885_c1_g2_i2*	Mitogen-activated protein kinase	9.72	—	Seed/pod
	*bs10dtrinity_dn12088_c3_g6_i6*	Mitogen-activated protein kinase	4.21	—	Seed/pod
	*bs10dtrinity_dn12088_c3_g6_i6*	Mitogen-activated protein kinase	—	5.55	Seed/pod
GA	*ab4dtrinity_dn7670_c0_g1_i4*	Transcription factor *PIF3*-like isoform	—	2.06	Days to flowering

### Simple Sequence Repeat Identification

**FIGURE 10:**
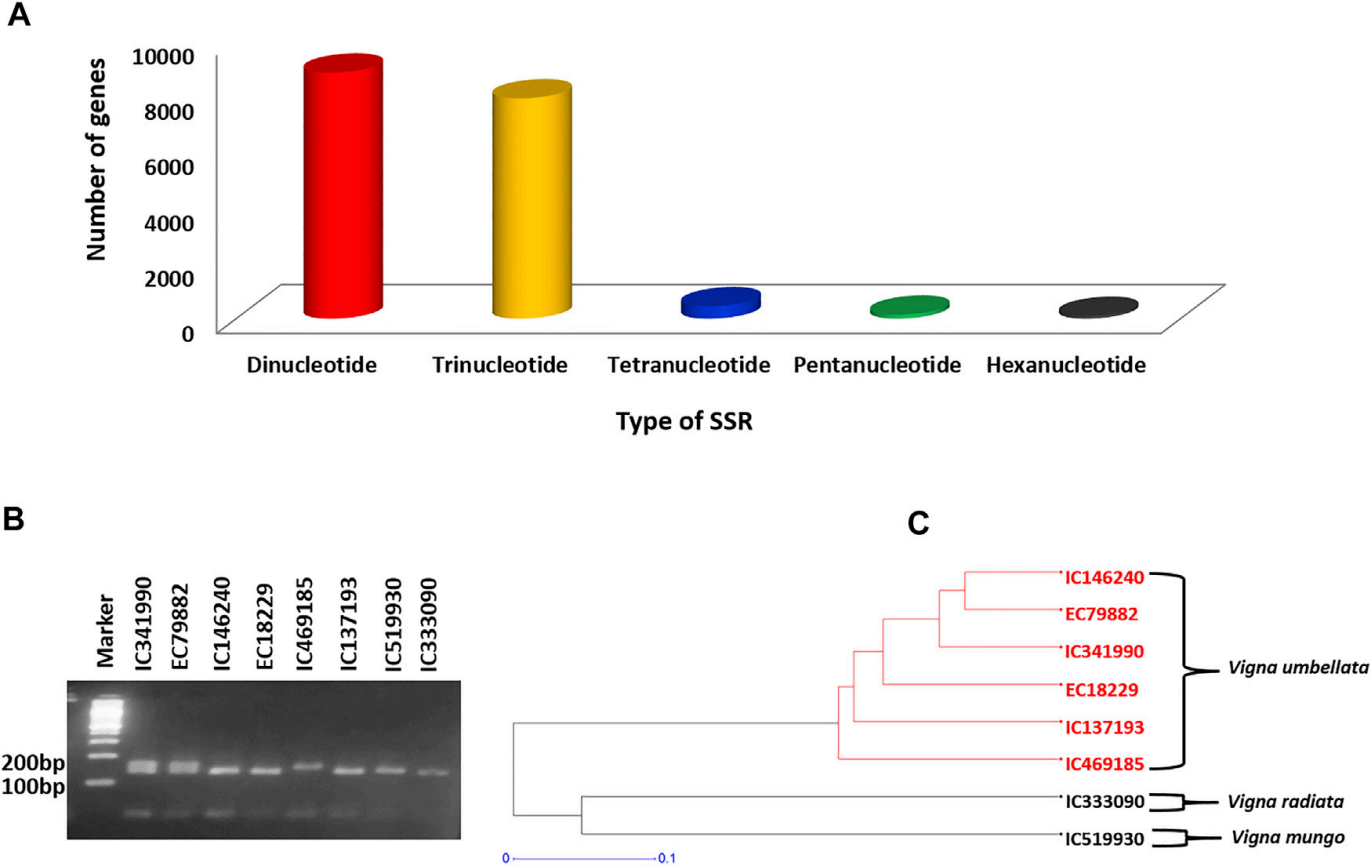
**(A)** Bar diagram representing the type and frequency of SSRs identified in ricebean using assembled transcripts. **(B)** SSR13 polymorphism on selected eight accessions of *Vigna* species. **(C)** Dendrogram representing the relationship distance among the eight accessions.

In this study, we used the MISA Perl script (http://pgrc.ipk-gatersleben.de/misa) to detect the microsatellites. Of the 288,393 transcripts generated in this study, 14,663 contained an SSR totaling 201,517,181 bp. Out of these 14,663 sequences, 2,317 sequences had more than a single SSR, and 1,487 had SSRs of different motifs (compound SSR). Dinucleotide repeat motifs were the most abundant among the five types of motifs, totaling 8,866 (50.67%). The second most abundant were trinucleotides totaling 7,938 (45.36%), followed by 448 tetranucleotides (2.56%), 145 pentanucleotides (0.82%), and 100 hexanucleotide motifs (0.57%) (Figure 10A). Similar results have been reported in the previous transcriptome published for ricebean varieties (Chen et al., 2016) as well as for other legume species including mungbean (Tian et al., 2016b), adzuki bean (Chankaew et al., 2014; Chen et al., 2015b), cowpea (Gupta et al., 2010), and chickpea (Choudhary et al., 2008).

The number of the given repeat unit of SSRs ranged from 5 to >10, and as the number of repeat units increased, the frequency of the given SSR structure progressively decreased (Supplementary Table S11). As for the two most abundant repeat motif types (di- and trinucleotides), the frequency of the AG/CT motif type accounted for 17.41% in dinucleotide repeat motifs, and the frequency of GAA/TTC was the most abundant motif type in the trinucleotide, accounting for 6.3%. A previous study on adzuki bean also showed a high frequency of AG motifs in dinucleotides (Chankaew et al., 2014).

### Simple Sequence Repeat Validation

To determine the polymorphism level of the identified EST-SSRs, the randomly selected 50 SSRs were evaluated in eight accessions of *Vigna* species including *V. umbellata* (6), *V. mungo* (1), and *V. radiata* (1) (Supplementary Table S12). From 50 pairs, 43 were successfully amplified, while seven pairs were not able to generate a PCR product (Supplementary Table S13). More than 85% of the SSR markers were successfully amplified, suggesting that the quality of our assembled transcripts was very high. The annealing temperatures of the primers ranged between 54 and 56°C. Out of these 43 SSR primer pairs, 26 pairs showed polymorphism (dinucleotide: 12, trinucleotide: 14) and the rest were monomorphic (Figure 10B; Table 5). A high polymorphism level (60.46%) of ricebean EST-SSRs was observed in the selected set of eight accessions which was higher than that from previous reports in other legume species including the chickpea (47.3%) (Nayak et al., 2010), mungbean (33%) (Chen et al., 2015b), black gram (58.2%) (Souframanien and Reddy, 2015), and adzuki bean (7.6%) (Chen et al., 2015a) while lower than common bean (71.3%) (Hanai et al., 2007), whereas when we considered only ricebean genotypes (six accessions), only 34.88% SSR markers were found polymorphic. We have also checked the cross-species transferability pattern and found that the transferability of ricebean–derived SSR markers was higher in *V. radiata* (73.08%) than in *V. mungo* (50%). Various studies depicted the importance of SSR cross-transferability in *Vigna* species including ricebean, mungbean, and cowpea (Pattanayak et al., 2019). Furthermore, the genetic distance among the accessions was determined, and we found two clusters, with six (*V. umbellata*) in the first cluster and two (*V. mungo* and *V. radiata*) in the second cluster, respectively (Figure 10C).

**TABLE 5:** List of 26 SSR markers that showed polymorphism in a set of eight accessions of *Vigna* species.

Transcript ID	Marker	Forward primer (5'->3′)	Reverse primer (5'->3′)	Annealing temp (°C)	Repeat motif	Allele size (bp)	No. of alleles
BB7DTRINITY_DN9136_c0_g1_i1	SSR2	ATG​ATC​GGA​CAC​TAG​GAG​AC	TTG​GCC​AAT​GTC​TAT​TTG​A	54	ATT(18)	150–160	2
BS7D1TRINITY_DN10554_c1_g1_i1	SSR3	ACG​CAC​AGT​TTC​ATG​GTT​A	ACA​ATC​TTC​AAC​CAC​ACT​CC	55	GAA(19)	100–130	4
BS4DTRINITY_DN12307_c0_g4_i3	SSR4	CAA​ACC​CAC​TAA​CCC​AAG​TA	ATG​AAA​ATG​CAA​ACA​CAC​TG	55	TAA(17)	140–150	2
CB4DTRINITY_DN13434_c0_g3_i2	SSR7	ATT​CCC​AGC​TTA​GGA​GAA​AC	TGG​ATT​TGT​TCT​TAA​TGG​TG	55	ATA(18)	140–170	2
AB4DTRINITY_DN10798_c2_g4_i2	SSR8	GTT​ATT​GGA​ATG​GAA​GAG​CA	CTT​CCG​ACA​ACA​ATT​CCT​T	55	GAA(16)	120–140	3
AS4DTRINITY_DN8490_c0_g1_i1	SSR9	CAA​CCG​GGT​AGA​GAA​AAG​TA	CTA​CCA​AGT​TGC​TTG​CTT​CT	54	AAT(22)	210–220	2
CB7DTRINITY_DN17452_c5_g1_i3	SSR11	ATG​GGT​TTC​CTA​TGA​ATT​TG	GCT​AAT​GAC​TCT​GCT​GTT​CC	55	TAA(11)	140–150	2
AB4DTRINITY_DN10727_c2_g3_i1	SSR12	GCT​AAT​GAC​TCT​GCT​GTT​CC	ATG​GGT​TTC​CTA​TGA​ATT​TG	55	TTA(11)	140–150	2
AS4DTRINITY_DN11798_c1_g2_i4	SSR13	GGG​AAA​ATG​TTA​CGG​AGT​TC	GTT​TTC​CCA​CCA​CAA​CTA​AC	56	TGG(12)	120–150	2
BS4DTRINITY_DN12006_c0_g8_i4	SSR14	CTG​GGA​AAC​TGA​GCA​GAT​AG	CAG​ATA​GTT​GCA​ATA​GCT​TGA​A	55	TAT(12)	170–190	3
CB7DTRINITY_DN16904_c1_g1_i5	SSR15	TTA​GAA​TTT​CCG​TTG​CTA​CC	CCC​TGA​AAG​AAG​TTT​GGA​AT	55	TAT(12)	170–180	2
BB7DTRINITY_DN17092_c1_g1_i3	SSR16	TTC​ACC​TCT​GAC​TGA​TCA​CA	CAA​GTC​TAA​TGC​ATC​CAC​CT	55	GAT(13)	160–180	3
BS7DTRINITY_DN11614_c2_g22_i1	SSR18	CTG​GGA​AAC​TGA​GCA​GAT​AG	CAG​ATA​GTT​GCA​ATA​GCT​TGA​A	55	TAT(12)	190–200	2
AS4DTRINITY_DN11028_c4_g1_i3	SSR24	CTG​GGA​AAC​TGA​GCA​GAT​AG	CAG​ATA​GTT​GCA​ATA​GCT​TGA​A	55	TAT(12)	190–200	2
BS4DTRINITY_DN11934_c2_g1_i5	SSR28	TTC​CAC​GTT​CTC​ACT​CTC​TT	GGA​ATC​CAT​TAC​TGT​GAA​CG	55	TC(37)	100–130	4
BS7DTRINITY_DN11790_c6_g2_i8	SSR30	CTC​TTC​TTA​GAG​CCA​AAC​CA	ACG​CCA​TGT​GTA​TGA​AGA​TT	55	CT(36)	100–120	3
BS7DTRINITY_DN3955_c0_g1_i2	SSR31	CGT​TTC​CTA​AGC​TTC​CTT​TA	GAG​AAG​CGA​AGA​AGA​AAG​GT	55	TC(35)	100–130	4
AB7DTRINITY_DN28298_c2_g1_i4	SSR32	CTA​CCA​GTG​GGT​TCG​TTT​AC	TCT​CTC​TTC​TCC​CCT​TAA​CC	55	GA(32)	130–160	4
AB4DTRINITY_DN10313_c1_g2_i7	SSR35	CAC​CCT​AAC​CTC​ATT​CTC​AG	GAC​AGC​AAG​AAG​GAG​AGA​GA	54	CT(48)	100–110	2
CB4DTRINITY_DN14022_c6_g1_i1	SSR37	TCA​CAA​AAC​CCT​AAA​ACT​CG	GGC​AGT​GTG​AAA​GAA​AGA​GA	55	TC(28)	200–220	3
CS4DTRINITY_DN11268_c2_g5_i1	SSR38	AAT​GTG​CTC​TTC​TTG​TTG​CT	ACC​GAT​GGA​ATA​ACC​AAA​C	55	TC(28)	100–110	2
BB7DTRINITY_DN13571_c0_g1_i4	SSR39	TTG​TGG​ATA​TAA​ACC​CAA​CC	GCT​CCT​CCG​CTC​TTC​TAT​TA	56	AG(28)	120–150	4
BB7DTRINITY_DN16958_c4_g1_i5	SSR40	TGA​TTA​ACT​GGG​TTC​TCT​GC	TTC​TAC​AAC​CAC​CCA​ATC​TC	55	AT(28)	110–130	3
BS7DTRINITY_DN11905_c0_g1_i6	SSR41	GGG​AGT​ATC​CAA​AGA​AAC​AA	AAT​CCA​CAC​ACA​AAT​GTG​AA	54	TC(30)	110–120	2
BB4DTRINITY_DN12047_c0_g1_i7	SSR42	GGA​ATC​CAT​TAC​TGT​GAA​CG	TTC​CAC​GTT​CTC​ACT​CTC​TT	55	GA(30)	110–140	4
CB7DTRINITY_DN16920_c1_g4_i9	SSR45	GTG​GGT​AAC​TAT​GCC​CTA​AGT	GGT​GAG​TGG​ATG​TGA​GAA​AG	55	TC(27)	110–120	2

SSR molecular markers on the basis of transcriptomes have become more promising and useful because of their high cross-species transferability, their high rate of amplification, and being reasonably cheap as compared with the SSR markers of non-transcribed regions (Hansen et al., 2008; Rai et al., 2013). Moreover, since they can easily expose variance in the expressed portion of the genome, it is possible to evaluate marker–trait association (MTA) and specific genomic regions stating important physio-agronomic traits (Kalia et al., 2011).

## Conclusion

The transcriptomic analysis in our study provided detailed insights into molecular processes and candidate genes controlling seed size and other seed development–related traits in ricebean. The MapMan and KEGG analyses confirmed that the phytohormone signaling pathways varied in both the contrasting expressions taken in this study and can therefore be the regulators of seed size as well as other seed development–related traits in ricebean. We hypothesize that the auxin, cytokinin, ethylene, and gibberellin signaling pathways interact cooperatively with one another, thereby modulating the expression of genes of seed development–related traits. Further research is required to identify key regulators/genes in determining seed size.

## Data Availability

The original contributions presented in the study are publicly available in NCBI under accession number PRJNA765494.

## References

[B1] Amkul K. Somta P. Laosatit K. Wang L. (2020). Identification of QTLs for Domestication-Related Traits in Zombi Pea [*Vigna vexillata* (L.) A. Rich], a Lost Crop of Africa. Front. Genet. 11, 803. 10.3389/FGENE.2020.00803 33193562 PMC7530282

[B2] Ashikari M. Sakakibara H. Lin S. Yamamoto T. Takashi T. Nishimura A. (2005). Cytokinin Oxidase Regulates Rice Grain Production. Science 309, 741–745. 10.1126/SCIENCE.1113373 15976269

[B3] Bae J. M. Kwak M. S. Noh S. A. Oh M. J. Kim Y. S. Shin J. S. (2014). Overexpression of Sweetpotato Expansin cDNA (IbEXP1) Increases Seed Yield in Arabidopsis. Transgenic Res. 23, 657–667. 10.1007/S11248-014-9804-1 24806537

[B4] Bai L. Chen Q. Jiang L. Lin Y. Ye Y. Liu P. (2019). Comparative Transcriptome Analysis Uncovers the Regulatory Functions of Long Noncoding RNAs in Fruit Development and Color Changes of *Fragaria pentaphylla* . Hortic. Res. 61 6, 1. 10.1038/s41438-019-0128-4 PMC6397888 30854215

[B5] Bartrina I. Jensen H. Novák O. Strnad M. Werner T. Schmülling T. (2017). Gain-of-Function Mutants of the Cytokinin Receptors AHK2 and AHK3 Regulate Plant Organ Size, Flowering Time and Plant Longevity. Plant Physiol. 173, 1783–1797. 10.1104/PP.16.01903 28096190 PMC5338655

[B6] Bartrina I. Otto E. Strnad M. Werner T. Schmülling T. (2011). Cytokinin Regulates the Activity of Reproductive Meristems, Flower Organ Size, Ovule Formation, and Thus Seed Yield in *Arabidopsis thaliana* . Plant Cell 23, 69–80. 10.1105/TPC.110.079079 21224426 PMC3051259

[B7] Benedito V. A. Torres-Jerez I. Murray J. D. Andriankaja A. Allen S. Kakar K. (2008). A Gene Expression Atlas of the Model Legume *Medicago truncatula* . Plant J. 55, 504–513. 10.1111/J.1365-313X.2008.03519.X 18410479

[B8] Benková E. Michniewicz M. Sauer M. Teichmann T. Seifertová D. Jürgens G. (2003). Local, Efflux-Dependent Auxin Gradients as a Common Module for Plant Organ Formation. Cell 115, 591–602. 10.1016/S0092-8674(03)00924-3 14651850

[B9] Bernardi J. Li Q.-B. Gao Y. Zhao Y. Battaglia R. Marocco A. (2016). The Auxin-Deficient Defective Kernel18 (dek18) Mutation Alters the Expression of Seed-Specific Biosynthetic Genes in Maize. J. Plant Growth Regul. 353 (35), 770–777. 10.1007/S00344-016-9581-6

[B10] Blakeslee J. J. Bandyopadhyay A. Peer W. A. Makam S. N. Murphy A. S. (2004). Relocalization of the PIN1 Auxin Efflux Facilitator Plays a Role in Phototropic Responses. Plant Physiol. 134, 28–31. 10.1104/PP.103.031690 14730061 PMC1540349

[B11] Borisjuk L. Rolletschek H. Radchuk R. Weschke W. Wobus U. Weber H. (2004). Seed Development and Differentiation: A Role for Metabolic Regulation. Plant Biol. 6, 375–386. 10.1055/s-2004-817908 15248120

[B12] Brinton J. Simmonds J. Minter F. Leverington-Waite M. Snape J. Uauy C. (2017). Increased Pericarp Cell Length Underlies a Major Quantitative Trait Locus for Grain Weight in Hexaploid Wheat. New Phytol. 215, 1026–1038. 10.1111/NPH.14624 28574181

[B13] Bromley J. R. Warnes B. J. Newell C. A. Thomson J. C. P. James C. M. Turnbull C. G. N. (2014). A Purine Nucleoside Phosphorylase in *Solanum tuberosum* L. (Potato) with Specificity for Cytokinins Contributes to the Duration of Tuber Endodormancy. Biochem. J. 458, 225–237. 10.1042/BJ20130792 24325449

[B14] Calzadilla P. I. Maiale S. J. Ruiz O. A. Escaray F. J. (2016). Transcriptome Response Mediated by Cold Stress in *Lotus japonicus* . Front. Plant Sci. 0, 374. 10.3389/FPLS.2016.00374 PMC4811897 27066029

[B15] Chankaew S. Isemura T. Isobe S. Kaga A. Tomooka N. Somta P. (2014). Detection of Genome Donor Species of Neglected Tetraploid Crop Vigna Reflexo-Pilosa (Créole Bean), and Genetic Structure of Diploid Species Based on Newly Developed EST-SSR Markers from Azuki Bean (*Vigna angularis*). PLoS One 9, e104990. 10.1371/JOURNAL.PONE.0104990 25153330 PMC4143246

[B16] Chapman E. J. Estelle M. (2009). Mechanism of Auxin-Regulated Gene Expression in Plants. Annu. Rev. Genet. 43, 265–285. 10.1146/annurev-genet-102108-134148 19686081

[B17] Chen H. Chen X. Tian J. Yang Y. Liu Z. Hao X. (2016). Development of Gene-Based SSR Markers in Rice Bean (*Vigna umbellata* L.) Based on Transcriptome Data. PLoS One 11, e0151040. 10.1371/JOURNAL.PONE.0151040 26950544 PMC4780709

[B18] Chen H. Liu L. Wang L. Wang S. Somta P. Cheng X. (2015a). Development and Validation of EST-SSR Markers from the Transcriptome of Adzuki Bean (*Vigna angularis*). PLoS One 10, 1–14. 10.1371/journal.pone.0131939 PMC4492930 26146990

[B19] Chen H. Wang L. Liu X. Hu L. Wang S. Cheng X. (2017). De Novo transcriptomic Analysis of Cowpea (*Vigna unguiculata* L. Walp) for Genic SSR Marker Development. BMC Genet. 18, 1–12. 10.1186/S12863-017-0531-5 28693419 PMC5504845

[B20] Chen H. Wang L. Wang S. Liu C. Blair M. W. Cheng X. (2015b). Transcriptome Sequencing of Mung Bean (*Vigna radiate* L.) Genes and the Identification of EST-SSR Markers. PLoS One 10, e0120273. 10.1371/JOURNAL.PONE.0120273 25830701 PMC4382333

[B21] Cheng Z. J. Zhao X. Y. Shao X. X. Wang F. Zhou C. Liu Y. G. (2014). Abscisic Acid Regulates Early Seed Development in Arabidopsis by ABI5-Mediated Transcription of Short Hypocotyl Under Blue1. Plant Cell 26, 1053–1068. 10.1105/TPC.113.121566 24619610 PMC4001368

[B22] Choudhary S. Sethy N. K. Shokeen B. Bhatia S. (2008). Development of Chickpea EST-SSR Markers and Analysis of Allelic Variation across Related Species. Theor. Appl. Genet. 118, 591–608. 10.1007/S00122-008-0923-Z 19020854

[B23] Christian M. Steffens B. Schenck D. Burmester S. Böttger M. Lüthen H. (2006). How Does Auxin Enhance Cell Elongation? Roles of Auxin-Binding Proteins and Potassium Channels in Growth Control. Plant Biol. 8, 346–352. 10.1055/S-2006-923965 16807827

[B24] Clevenger J. Chu Y. Scheffler B. Ozias-Akins P. (2016). A Developmental Transcriptome Map for Allotetraploid *Arachis hypogaea* . Front. Plant Sci. 0, 1446. 10.3389/FPLS.2016.01446 PMC5043296 27746793

[B25] Daele I. V. Gonzalez N. Ilse I. V. P. (2012). A Comparative Study of Seed Yield Parameters in *Arabidopsis thaliana* Mutants and Transgenics. Plant Biol. J. 10, 488–500. 10.1111/j.1467-7652.2012.00687.x 22332878

[B26] Desbrosses G. J. Stougaard J. (2011). Root Nodulation: A Paradigm for How Plant-Microbe Symbiosis Influences Host Developmental Pathways. Cell Host Microbe 10, 348–358. 10.1016/J.CHOM.2011.09.005 22018235

[B27] Fornara F. Panigrahi K. C. S. Gissot L. Sauerbrunn N. Rühl M. Jarillo J. A. (2009). Arabidopsis DOF Transcription Factors Act Redundantly to Reduce CONSTANS Expression and are Essential for a Photoperiodic Flowering Response. Dev. Cell 17, 75–86. 10.1016/J.DEVCEL.2009.06.015 19619493

[B28] Garg R. Patel R. K. Jhanwar S. Priya P. Bhattacharjee A. Yadav G. (2011). Gene Discovery and Tissue-specific Transcriptome Analysis in Chickpea with Massively Parallel Pyrosequencing and Web Resource Development. Plant Physiol. 156, 1661–1678. 10.1104/pp.111.178616 21653784 PMC3149962

[B29] Geng X. Dong N. Wang Y. Li G. Wang L. Guo X. (2018). Correction: RNA-Seq Transcriptome Analysis of the Immature Seeds of Two Brassica Napus Lines with Extremely Different Thousand-Seed Weight to Identify the Candidate Genes Related to Seed Weight. PLoS ONE 13, 1. 10.1371/journal.pone.0218914 PMC5790231 29381708

[B30] Glover J. Grelon M. Craig S. Chaudhury A. Dennis E. (1998). Cloning and Characterization of MS5 from Arabidopsis : A Gene Critical in Male Meiosis. Plant J. 15, 345–356. 10.1046/J.1365-313X.1998.00216.X 9750346

[B31] González A. M. Yuste-Lisbona F. J. Weller J. Vander Schoor J. K. Lozano R. Santalla M. (2021). Characterization of QTL and Environmental Interactions Controlling Flowering Time in Andean Common Bean (*Phaseolus vulgaris* L.). Front. Plant Sci. 11, 599462. 10.3389/FPLS.2020.599462 33519852 PMC7840541

[B32] Gu D. Andreev K. Dupre E. M. (2021). Major Trends in Population Growth Around the World. China CDC Wkly 3, 604–613. 10.46234/ccdcw2021.160 34594946 PMC8393076

[B33] Guo B. Wei Y. Xu R. Lin S. Luan H. Lv C. (2016). Genome-Wide Analysis of APETALA2/Ethylene-Responsive Factor (AP2/ERF) Gene Family in Barley (*Hordeum vulgare* L.). PLoS One 11, e0161322. 10.1371/JOURNAL.PONE.0161322 27598245 PMC5012588

[B34] Gupta P. Singh R. Malhotra S. Boora K. S. Singal H. R. (2010). Characterization of Seed Storage Proteins in High Protein Genotypes of Cowpea [*Vigna unguiculata* (L.) Walp.]. Physiol. Mol. Biol. Plants 161, 53–58. 10.1007/S12298-010-0007-9 PMC3550628 23572954

[B35] Hanai L. R. De Campos T. Camargo L. E. A. Benchimol L. L. De Souza A. P. Melotto M. (2007). Development, Characterization, and Comparative Analysis of Polymorphism at Common Bean SSR Loci Isolated from Genic and Genomic Sources. Genome 50, 266–277. 10.1139/G07-007 17502900

[B36] Hansen B. G. Halkier B. A. Kliebenstein D. J. (2008). Identifying the Molecular Basis of QTLs: eQTLs Add a New Dimension. Trends Plant Sci. 13, 72–77. 10.1016/J.TPLANTS.2007.11.008 18262820

[B37] Hayama R. Yokoi S. Tamaki S. Yano M. Shimamoto K. (2003). Adaptation of Photoperiodic Control Pathways Produces Short-Day Flowering in Rice. Nature 422, 719–722. 10.1038/nature01549 12700762

[B38] Hayashi K. (2012). The Interaction and Integration of Auxin Signaling Components. Plant Cell Physiol. 53, 965–975. 10.1093/PCP/PCS035 22433459

[B39] Hentrich M. Sánchez-Parra B. Alonso M.-M. P. Loba V. C. Carrillo L. Vicente-Carbajosa J. (2013). YUCCA8 and YUCCA9 Overexpression Reveals a Link between Auxin Signaling and Lignification through the Induction of Ethylene Biosynthesis, Plant Signal. Behav. 8, e26363. 10.4161/psb.26363 24022251 PMC4106514

[B40] Herridge R. P. Day R. C. Baldwin S. Macknight R. C. (2011). Rapid Analysis of Seed Size in Arabidopsis for Mutant and QTL Discovery. Plant Methods 71 (7), 1. 10.1186/1746-4811-7-3 PMC3046896 21303553

[B41] Heyl A. Riefler M. Romanov G. A. Schmülling T. (2012). Properties, Functions and Evolution of Cytokinin Receptors. Eur. J. Cell Biol. 91, 246–256. 10.1016/J.EJCB.2011.02.009 21561682

[B42] Heyl A. Schmülling T. (2003). Cytokinin Signal Perception and Transduction. Curr. Opin. Plant Biol. 6, 480–488. 10.1016/S1369-5266(03)00087-6 12972049

[B43] Hiz M. C. Canher B. Niron H. Turet M. (2014). Transcriptome Analysis of Salt Tolerant Common Bean (*Phaseolus vulgaris* L.) under Saline Conditions. PLoS One 9, e92598. 10.1371/JOURNAL.PONE.0092598 24651267 PMC3961409

[B44] Hornett E. A. Wheat C. W. (2012). Quantitative RNA-Seq Analysis in Non-Model Species: Assessing Transcriptome Assemblies as a Scaffold and the Utility of Evolutionary Divergent Genomic Reference Species. BMC Genom. 13, 361. 10.1186/1471-2164-13-361 PMC3469347 22853326

[B45] Huang J. Deng J. Shi T. Chen Q. Liang C. Meng Z. (2017). Global Transcriptome Analysis and Identification of Genes Involved in Nutrients Accumulation during Seed Development of rice Tartary Buckwheat (*Fagopyrum tararicum*). Sci. Rep. 71, 1–14. 10.1038/s41598-017-11929-z PMC5603606 28924217

[B46] Huang R. Jiang L. Zheng J. Wang T. Wang H. Huang Y. (2013). Genetic Bases of rice Grain Shape: So many Genes, So Little Known. Trends Plant Sci. 18, 218–226. 10.1016/J.TPLANTS.2012.11.001 23218902

[B47] Hwang I. Sheen J. Müller B. (2012). Cytokinin Signaling Networks. Annu. Rev. Plant Biol. 63, 353. 10.1146/annurev-arplant-042811-105503 22554243

[B48] Jain M. Kaur N. Garg R. Thakur J. K. Tyagi A. K. Khurana J. P. (20052005). Structure and Expression Analysis of Early Auxin-Responsive Aux/IAA Gene Family in Rice (*Oryza sativa*). Funct. Integr. Genom. 61 6, 47–59. 10.1007/S10142-005-0005-0 16200395

[B49] Jiang H. Li Y. Qin H. Li Y. Qi H. Li C. (2018). Identification of Major QTLs Associated with First Pod Height and Candidate Gene Mining in Soybean. Front. Plant Sci. 0, 1280. 10.3389/FPLS.2018.01280 PMC6157441 30283463

[B50] Jones S. I. Vodkin L. O. (2013). Using RNA-Seq to Profile Soybean Seed Development from Fertilization to Maturity. PLoS One 8. 10.1371/journal.pone.0059270 PMC3598657 23555009

[B51] Kalia R. K. Rai M. K. Kalia S. Singh R. Dhawan A. K. (2011). Microsatellite Markers: An Overview of the Recent Progress in Plants. Euphytica 177, 309–334. 10.1007/s10681-010-0286-9

[B52] Kalluri U. C. DiFazio S. P. Brunner A. M. Tuskan G. A. (2007). Genome-Wide Analysis of Aux/IAA and ARF Gene Families in Populus trichocarpa. BMC Plant Biol. 71 (7), 1. 10.1186/1471-2229-7-59 PMC2174922 17986329

[B53] Katoch R. (2013). Nutritional Potential of Rice Bean (*Vigna umbellata*): An Underutilized Legume. J. Food Sci. 78, 8–16. 10.1111/j.1750-3841.2012.02989.x 23278402

[B54] Kaul T. Eswaran M. Thangaraj A. Meyyazhagan A. Nehra M. Raman N. M. (2019). Rice Bean (*Vigna umbellata*) Draft Genome Sequence: Unravelling the Late Flowering and Unpalatability Related Genomic Resources for Efficient Domestication of This Underutilized Crop. bioRxiv, 816595. 10.1101/816595

[B55] Komiya R. Yokoi S. Shimamoto K. (2009). A Gene Network for Long-Day Flowering Activates RFT1 Encoding a mobile Flowering Signal in Rice. Development 136, 3443–3450. 10.1242/DEV.040170 19762423

[B56] Kumar A. Patel J. S. Meena V. S. Srivastava R. (2019). Recent Advances of PGPR Based Approaches for Stress Tolerance in Plants for Sustainable Agriculture. Biocatal. Agric. Biotechnol. 20, 101271. 10.1016/J.BCAB.2019.101271

[B57] Leyser O. (2003). Molecular Genetics of Auxin Signaling. Annu. Rev. Plant Biol. 53, 377. 10.1146/annurev.arplant.53.100301.135227 12221981

[B58] Li H. Lv Q. Deng J. Huang J. Cai F. Liang C. (2019a). Transcriptome Analysis Reveals Key Seed-Development Genes in Common Buckwheat (*Fagopyrum esculentum*). Int. J. Mol. Sci. 20, 4303. 10.3390/ijms20174303 PMC6747174 31484314

[B59] Li N. Wang Y. Lu J. Liu C. (2019b). Genome-Wide Identification and Characterization of the ALOG Domain Genes in Rice. Int. J. Genomics 2019, 2146391. 10.1155/2019/2146391 30923712 PMC6409076

[B60] Liu N. Zhang G. Xu S. Mao W. Hu Q. Gong Y. (2015). Comparative Transcriptomic Analyses of Vegetable and Grain Pea (*Pisum sativum* L.) Seed Development. Front. Plant Sci. 0, 1039. 10.3389/FPLS.2015.01039 PMC4658420 26635856

[B61] Liu T. Zhu S. Tang Q. Chen P. Yu Y. Tang S. (2013). *De Novo* Assembly and Characterization of Transcriptome Using Illumina Paired-End Sequencing and Identification of CesA Gene in Ramie (*Boehmeria nivea* L. Gaud ). BMC Genomics 14, 125. 10.1186/1471-2164-14-125 23442184 PMC3610122

[B62] Lo S. Muñoz-Amatriaín M. Boukar O. Herniter I. Cisse N. Guo Y.-N. (2018). Identification of QTL Controlling Domestication-Related Traits in Cowpea (*Vigna unguiculata* L. Walp). Sci. Rep. 81 8, 1–9. 10.1038/s41598-018-24349-4 PMC5908840 29674702

[B63] Lo S. Muñoz-Amatriaín M. Hokin S. A. Cisse N. Roberts P. A. Farmer A. D. (2019). A Genome-wide Association and Meta-Analysis Reveal Regions Associated with Seed Size in Cowpea [*Vigna unguiculata* (L.) Walp]. Theor. Appl. Genet. 132, 3079–3087. 10.1007/S00122-019-03407-Z 31367839 PMC6791911

[B64] Lohar D. P. Schaff J. E. Laskey J. G. Kieber J. J. Bilyeu K. D. Bird D. M. (2004). Cytokinins Play Opposite Roles in Lateral Root Formation, and Nematode and Rhizobial Symbioses. Plant J. 38, 203–214. 10.1111/J.1365-313X.2004.02038.X 15078325

[B65] Lonardi S. Muñoz-Amatriaín M. Liang Q. Shu S. Wanamaker S. I. Lo S. (2019). The Genome of Cowpea (*Vigna unguiculata* [L.] Walp.). Plant J. 98, 767–782. 10.1111/TPJ.14349 31017340 PMC6852540

[B66] Ma M. Wang Q. Li Z. Cheng H. Li Z. Liu X. (2015). Expression of TaCYP78A3, a Gene Encoding Cytochrome P450 CYP78A3 Protein in Wheat (*Triticum aestivum* L.), Affects Seed Size. Plant J. 83, 312–325. 10.1111/TPJ.12896 26043144

[B67] Mahto A. Mathew I. E. Agarwal P. (2017). Decoding the Transcriptome of Rice Seed During Development. Adv. Seed Biol., 25 10.5772/INTECHOPEN.70574

[B68] Möller B. Weijers D. (2009). Auxin Control of Embryo Patterning. Cold Spring Harb. Perspect. Biol. 1, a001545. 10.1101/CSHPERSPECT.A001545 20066117 PMC2773644

[B69] Muñoz M. Calderini D. F. (2015). Volume, Water Content, Epidermal Cell Area, and XTH5 Expression in Growing Grains of Wheat across Ploidy Levels. F. Crop Res. 173, 30–40. 10.1016/J.FCR.2014.12.010

[B70] Nakashima K. Yamaguchi-Shinozaki K. (2013). ABA Signaling in Stress-Response and Seed Development. Plant Cell Rep. 327 (32), 959–970. 10.1007/S00299-013-1418-1 23535869

[B71] Nakayama A. Park S. Zheng-Jun X. Nakajima M. Yamaguchi I. (2002). Immunohistochemistry of Active Gibberellins and Gibberellin-Inducible α-Amylase in Developing Seeds of Morning Glory. Plant Physiol. 129, 1045–1053. 10.1104/PP.010921 12114559 PMC166499

[B72] Nayak S. N. Zhu H. Varghese N. Datta S. Choi H. K. Horres R. (2010). Integration of Novel SSR and Gene-Based SNP Marker Loci in the Chickpea Genetic Map and Establishment of New Anchor Points with Medicago Truncatula Genome. Theor. Appl. Genet. 120, 1415–1441. 10.1007/s00122-010-1265-1 20098978 PMC2854349

[B73] Nelson S. K. Ariizumi T. Steber C. M. (2017). Biology in the Dry Seed: Transcriptome Changes Associated with Dry Seed Dormancy and Dormancy Loss in the Arabidopsis GA-insensitive Sleepy1-2 Mutant. Front. Plant Sci. 8, 1–21. 10.3389/fpls.2017.02158 29312402 PMC5744475

[B74] Ozga J. A. Yu J. Reinecke D. M. (2003). Pollination-, Development-, and Auxin-specific Regulation of Gibberellin 3β-Hydroxylase Gene Expression in Pea Fruit and Seeds. Plant Physiol. 131, 1137–1146. 10.1104/PP.102.015974 12644664 PMC166877

[B75] Parry G. Calderon-Villalobos L. I. Prigge M. Peret B. Dharmasiri S. Itoh H. (2009). Complex Regulation of the TIR1/AFB Family of Auxin Receptors. Proc. Natl. Acad. Sci. 106, 22540–22545. 10.1073/PNAS.0911967106 20018756 PMC2799741

[B76] Pattanayak A. Roy S. Sood S. Iangrai B. Banerjee A. Gupta S. (2019). Rice Bean: A Lesser Known Pulse with Well-Recognized Potential. Planta 250, 873–890. 10.1007/s00425-019-03196-1 31134340

[B77] Pazhamala L. T. Agarwal G. Bajaj P. Kumar V. Kulshreshtha A. Saxena R. K. (2016). Deciphering Transcriptional Programming during Pod and Seed Development Using RNA-Seq in Pigeonpea (*Cajanus cajan*). PLoS One 11, e0164959. 10.1371/JOURNAL.PONE.0164959 27760186 PMC5070767

[B78] Peng L. Qian L. Wang M. Liu W. Song X. Cheng H. (2021). Comparative Transcriptome Analysis during Seeds Development between Two Soybean Cultivars. PeerJ 9, 1–20. 10.7717/peerj.10772 PMC7931715 33717671

[B79] Pradhan S. Bandhiwal N. Shah N. Kant C. Gaur R. Bhatia S. (2014). Global Transcriptome Analysis of Developing Chickpea (*Cicer arietinum* L.) Seeds. Front. Plant Sci. 5, 1–14. 10.3389/fpls.2014.00698 PMC4267183 25566273

[B80] Qi Z. Zhang Z. Wang Z. Yu J. Qin H. Mao X. (2018). Meta-analysis and Transcriptome Profiling Reveal Hub Genes for Soybean Seed Storage Composition during Seed Development. Plant Cell Environ. 41, 2109–2127. 10.1111/pce.13175 29486529

[B81] Rai M. K. Phulwaria M. Shekhawat N. S. (2013). Transferability of Simple Sequence Repeat (SSR) Markers Developed in Guava (*Psidium guajava* L.) to Four Myrtaceae Species. Mol. Biol. Rep. 40, 5067–5071. 10.1007/s11033-013-2608-1 23657599

[B82] Raizada A. Jegadeesan S. (2020). Comparative Transcriptomic Analysis Revealed Complex 2 Molecular Mechanisms Underlying Pests, Pathogens Resistance 3 and Seed Development in Wild and Cultivated Blackgram. bioRxiv

[B83] Rashotte A. M. Poupart J. Waddell C. S. Muday G. K. (2003). Transport of the Two Natural Auxins, Indole-3-Butyric Acid and Indole-3-Acetic Acid, in Arabidopsis. Plant Physiol. 133, 761–772. 10.1104/PP.103.022582 14526119 PMC219050

[B84] Reinhardt D. Mandel T. Kuhlemeier C. (2000). Auxin Regulates the Initiation and Radial Position of Plant Lateral Organs. Plant Cell 12, 507–518. 10.1105/TPC.12.4.507 10760240 PMC139849

[B85] Riefler M. Novak O. Strnad M. Schmülling T. (2006). Arabidopsis Cytokinin Receptor Mutants Reveal Functions in Shoot Growth, Leaf Senescence, Seed Size, Germination, Root Development, and Cytokinin Metabolism. Plant Cell 18, 40–54. 10.1105/TPC.105.037796 16361392 PMC1323483

[B86] Robert C. Noriega A. Tocino Á. Cervantes E. (2008). Morphological Analysis of Seed Shape in *Arabidopsis thaliana* Reveals Altered Polarity in Mutants of the Ethylene Signaling Pathway. J. Plant Physiol. 165, 911–919. 10.1016/J.JPLPH.2007.10.005 18155318

[B87] Ruuska S. A. Schwender J. Ohlrogge J. B. (2004). The Capacity of Green Oilseeds to Utilize Photosynthesis to Drive Biosynthetic Processes. Plant Physiol. 136, 2700–2709. 10.1104/PP.104.047977 15347783 PMC523334

[B88] Schmutz J. McClean P. E. Mamidi S. Wu G. A. Cannon S. B. Grimwood J. (2014). A Reference Genome for Common Bean and Genome-wide Analysis of Dual Domestications. Nat. Genet. 467 46, 707–713. 10.1038/ng.3008 PMC7048698 24908249

[B89] Schruff M. C. Spielman M. Tiwari S. Adams S. Fenby N. Scott R. J. (2006). The Auxin Response Factor 2 Gene of Arabidopsis Links Auxin Signalling, Cell Division, and the Size of Seeds and Other Organs. Development 133, 251–261. 10.1242/DEV.02194 16339187

[B90] Shen C. Bai Y. Wang S. Zhang S. Wu Y. Chen M. (2010). Expression Profile of PIN, AUX/LAX and PGP Auxin Transporter Gene Families in Sorghumbicolorunder Phytohormone and Abiotic Stress. FEBS J. 277, 2954–2969. 10.1111/J.1742-4658.2010.07706.X 20528920

[B91] Shlamovitz N. Ziv M. Zamski E. (19951995). Light, Dark and Growth Regulator Involvement in Groundnut (*Arachis hypogaea* L.) Pod Development. Plant Growth Regul. 16, 37–42. 10.1007/BF00040505

[B92] Singh D. P. Jermakow A. M. Swain S. M. (2002). Gibberellins Are Required for Seed Development and Pollen Tube Growth in Arabidopsis. Plant Cell 14, 3133–3147. 10.1105/TPC.003046 12468732 PMC151207

[B93] Sinha P. Bajaj P. Pazhamala L. T. Nayak S. N. Pandey M. K. Chitikineni A. (2020). *Arachis hypogaea* Gene Expression Atlas for Fastigiata Subspecies of Cultivated Groundnut to Accelerate Functional and Translational Genomics Applications. Plant Biotechnol. J. 18, 2187–2200. 10.1111/PBI.13374 32167667 PMC7589347

[B94] Souframanien J. Reddy K. S. (2015). De Novo Assembly, Characterization of Immature Seed Transcriptome and Development of Genic-SSR Markers in Black Gram [*Vigna mungo* (L.) Hepper]. PLoS One 10, e0128748. 10.1371/JOURNAL.PONE.0128748 26042595 PMC4456365

[B95] Suanum W. Somta P. Kongjaimun A. Yimram T. Kaga A. Tomooka N. (2016). Co-Localization of QTLs for Pod Fiber Content and Pod Shattering in F 2 and Backcross Populations between Yardlong Bean and Wild Cowpea. Mol. Breed. 36, 1–11. 10.1007/S11032-016-0505-8

[B96] Sudheesh S. Sawbridge T. I. Cogan N. O. Kennedy P. Forster J. W. Kaur S. (2015). *De Novo* Assembly and Characterisation of the Field Pea Transcriptome Using RNA-Seq. BMC Genomics 16, 611. 10.1186/S12864-015-1815-7 26275991 PMC4537571

[B97] Takahashi T. Gasch A. Nishizawa N. Chua N. H. (1995). The DIMINUTO Gene of Arabidopsis is Involved in Regulating Cell Elongation. Genes Dev. 9, 97–107. 10.1101/GAD.9.1.97 7828854

[B98] Takahashi Y. Sakai H. Yoshitsu Y. Muto C. Anai T. Pandiyan M. (2019). Domesticating Vigna Stipulacea: A Potential Legume Crop with Broad Resistance to Biotic Stresses. Front. Plant Sci. 10, 1607. 10.3389/FPLS.2019.01607 31867036 PMC6909428

[B99] Tamaki S. Matsuo S. Hann L. W. Yokoi S. Shimamoto K. (2007). Hd3a Protein is a Mobile Flowering Signal in Rice. Science 316, 1033–1036. 10.1126/science.1141753 17446351

[B100] Tang H. Cuevas H. E. Das S. Sezen U. U. Zhou C. Guo H. (2013). Seed Shattering in a Wild Sorghum is Conferred by a Locus Unrelated to Domestication. Proc. Natl. Acad. Sci. 110, 15824–15829. 10.1073/PNAS.1305213110 24019506 PMC3785776

[B101] Tarkowski P. Tarkowská D. Novák O. Mihaljević S. Magnus V. Strnad M. (2006). Cytokinins in the Perianth, Carpels, and Developing Fruit of *Helleborus niger* L. J. Exp. Bot. 57, 2237–2247. 10.1093/JXB/ERJ190 16766598

[B102] Tian J. Isemura T. Kaga A. Vaughan D. A. Tomooka N. McIntyre C. L. (2013). Genetic Diversity of the rice Bean (Vigna Umbellata) Genepool as Assessed by SSR Markers. Genome 56, 717–727. 10.1139/gen-2013-0118 24433207

[B103] Tian X. Li S. Liu Y. Liu X. (2016a). Transcriptomic Profiling Reveals Metabolic and Regulatory Pathways in the Desiccation Tolerance of Mungbean (*Vigna radiata* [L.] R. Wilczek). Front. Plant Sci. 7, 1–12. 10.3389/fpls.2016.01921 28066476 PMC5174128

[B104] Tian Y. Zhang M. Hu X. Wang L. Dai J. Xu Y. (2016b). Over-Expression of CYP78A98, a Cytochrome P450 Gene from *Jatropha curcas* L., Increases Seed Size of Transgenic Tobacco. Electron. J. Biotechnol. 19, 15–22. 10.1016/j.ejbt.2015.11.001

[B105] Walton L. J. Kurepin L. V. Yeung E. C. Shah S. Emery R. J. N. Reid D. M. (2012). Ethylene Involvement in Silique and Seed Development of Canola, *Brassica napus* L. Plant Physiol. Biochem. 58, 142–150. 10.1016/J.PLAPHY.2012.06.016 22809685

[B106] Wan L. Li B. Lei Y. Yan L. Ren X. Chen Y. (2017). Mutant Transcriptome Sequencing Provides Insights into Pod Development in Peanut (*Arachis hypogaea* L.). Front. Plant Sci. 0, 1900. 10.3389/FPLS.2017.01900 PMC5684126 29170673

[B107] Watcharatpong P. Kaga A. Chen X. Somta P. (2020). Narrowing Down a Major QTL Region Conferring Pod Fiber Contents in Yardlong Bean (*Vigna unguiculata*), a Vegetable Cowpea. Genes 11, 363. 10.3390/GENES11040363 PMC7230914 32230893

[B108] Werner T. Holst K. Pörs Y. Guivarc’h A. Mustroph A. Chriqui D. (2008). Cytokinin Deficiency Causes Distinct Changes of Sink and Source Parameters in Tobacco Shoots and Roots. J. Exp. Bot. 59, 2659–2672. 10.1093/JXB/ERN134 18515826 PMC2486470

[B109] Xiang J. Tang S. Zhi H. Jia G. Wang H. Diao X. (2017). Loose Panicle1 Encoding a Novel WRKY Transcription Factor, Regulates Panicle Development, Stem Elongation, and Seed Size in Foxtail Millet [*Setaria italica* (L.) P. Beauv.]. PLoS One 12, e0178730. 10.1371/JOURNAL.PONE.0178730 28570666 PMC5453597

[B110] Xu W. Huang W. (20172017). Calcium-Dependent Protein Kinases in Phytohormone Signaling Pathways. Int. J. Mol. Sci. 18, 2436. 10.3390/IJMS18112436, PMC5713403 29156607

[B111] Ye J. Fang L. Zheng H. Zhang Y. Chen J. Zhang Z. (2006). WEGO: A Web Tool for Plotting GO Annotations. Nucleic Acids Res. 34, W293–W297. 10.1093/NAR/GKL031 16845012 PMC1538768

[B112] Yi F. Gu W. Chen J. Song N. Gao X. Zhang X. (2019). High Temporal-Resolution Transcriptome Landscape of Early maize Seed Development. Plant Cell 31, 974–992. 10.1105/tpc.18.00961 30914497 PMC6533015

[B113] Zhang D. F. Zhang N. Zhong T. Wang C. Xu M. L. Ye J. R. (2016). Identification and Characterization of the GH3 Gene Family in Maize. J. Integr. Agric. 15, 249–261. 10.1016/S2095-3119(15)61076-0

[B114] Zhong R. Kays S. J. Schroeder B. P. Ye Z.-H. (2002). Mutation of a Chitinase-Like Gene Causes Ectopic Deposition of Lignin, Aberrant Cell Shapes, and Overproduction of Ethylene. Plant Cell 14, 165–179. 10.1105/TPC.010278 11826306 PMC150558

[B115] Zhou Y. Lu D. Li C. Luo J. Zhu B.-F. Zhu J. (2012). Genetic Control of Seed Shattering in Rice by the APETALA2 Transcription Factor Shattering Abortion1. Plant Cell 24, 1034–1048. 10.1105/TPC.111.094383 22408071 PMC3336138

[B116] Zhu W. Chen X. Li H. Zhu F. Hong Y. Varshney R. K. (2014). Comparative Transcriptome Analysis of Aerial and Subterranean Pods Development Provides Insights into Seed Abortion in Peanut. Plant Mol. Biol. 85, 395–409. 10.1007/s11103-014-0193-x 24793121 PMC4152868

[B117] Zhu Z. Chen H. Xie K. Liu C. Li L. Liu L. (2020). Characterization of Drought-Responsive Transcriptome during Seed Germination in Adzuki Bean (*Vigna angularis* L.) by PacBio SMRT and Illumina Sequencing. Front. Genet. 11, 1–14. 10.3389/fgene.2020.00996 33110419 PMC7489039

[B118] Ziv M. Kahana O. R. A. (1988). “Photo Morphogenetic Response of the Embryo of Early-Stage Embryos Depended on the Presence of an Intact Suspensor.in” Injury to the Sus- Pensor at the Globular and Early Heart Stage Caused Abortion of Embryos Cultured *In Vitro* . bioRxiv. 57, 159–164.

