# Supplementary material for: Transcriptome Analysis Reveals Key Pathways and Candidate Genes Controlling Seed Development and Size in Ricebean (Vigna umbellata)
Source: Front Genet. 2022 Jan 21;12:791355. doi: 10.3389/fgene.2021.791355 (PMC8815620; doi:10.3389/fgene.2021.791355)
Supplement: Supplementary file 2 [file Table11.pdf]

Table S11: Summary of the number of repeat units in ricebean SSRs loci.

Motif Length	Repeat Numbers						Total Motifs
	5	6	7	8	9	>10	
**Di-nucleotide**	–	3094	1676	1123	796	2177	8866
**Tri-nucleotide**	4572	1843	827	478	79	139	7938
**Tetra-nucleotide**	294	117	13	8	4	12	448
**Penta-nucleotide**	102	7	13	7	7	9	145
**Hexa-nucleotide**	47	22	13	10	2	6	100
**Total**	5015	5083	2542	1626	888	2343	17497
