# Supplementary material for: Transcriptome Analysis Reveals Key Pathways and Candidate Genes Controlling Seed Development and Size in Ricebean (Vigna umbellata)
Source: Front Genet. 2022 Jan 21;12:791355. doi: 10.3389/fgene.2021.791355 (PMC8815620; doi:10.3389/fgene.2021.791355)
Supplement: Supplementary file 7 [file Table12.pdf]

Table S12: Details of the Vigna species accessions used in this study.

S. No.	Species	Accession	Source of collection/origin
1	*Vigna umbellata*	IC341990	India
2	*V. umbellata*	EC97882	USA
3	*V. umbellata*	IC146240	India
4	*V. umbellata*	EC18229	Nepal
5	*V. umbellata*	IC469185	India
6	*V. umbellata*	IC137193	India
7	*Vigna mungo*	IC519930	India
8	*Vigna radiata*	IC333090	India
