# Supplementary material for: Transcriptome Analysis Reveals Key Pathways and Candidate Genes Controlling Seed Development and Size in Ricebean (Vigna umbellata)
Source: Front Genet. 2022 Jan 21;12:791355. doi: 10.3389/fgene.2021.791355 (PMC8815620; doi:10.3389/fgene.2021.791355)
Supplement: Supplementary file 13 [file Table10.pdf]

Table S10: List of domains identified by Pfam for Ricebean candidate genes

Trait Name	Ricebean Candidate Gene ID	Best hit (Blast search)	Annotation	Ricebean Candidate Gene domains found by Pfam
Seed size	CS4DTRINITY_DN10996_c2_g4_i3	Vigun08g217000	histidine kinase 2	CHASE; HATPase_c; HisKA; Response_reg
	CS4DTRINITY_DN11621_c1_g7_i3	Vigun11g191300	Delta(24)-sterol reductase (EC 1.3.1.72)	FAD_binding
	CS4DTRINITY_DN18119_c0_g1_i1	Vigun05g039600	phosphate transporter PHO1 homolog 9-like	EXS
	CS4DTRINITY_DN25613_c0_g1_i1	Vigun09g215300	WRKY domain-containing protein (WRKY40)	WRKY
Seed/pod	AB4DTRINITY_DN10550_c1_g1_i11	AT1G30490	Uncharacterized protein	3Beta_HSD; Epimerase; GDP_Man_Dehyd; Homeobox; MEKHLA; NAD_binding_10; START
	AB4DTRINITY_DN11214_c0_g9_i1	AT1G12260|AT1G12260.1	NAC domain-containing protein (NAC37)	NAM
	AB7DTRINITY_DN14585_c0_g1_i2	AT1G34430|AT1G34430.1	Dihydrolipoamide acetyltransferase component of pyruvate dehydrogenase complex (EC 2.3.1.-)	2-oxoacid_dh; Biotin_lipoyl; E3_binding
	AB7DTRINITY_DN28783_c0_g1_i2	AT2G47470|AT2G47470.1; AT2G47470.4;AT2G47470.3;AT2G47470.2	Protein disulfide-isomerase (EC 5.3.4.1)	AhpC-TSA; OST3_OST6; Redoxin; Thioredoxin; Thioredoxin_2
	AB7DTRINITY_DN29885_c1_g2_i2	AT3G45640|AT3G45640.1	Mitogen-activated protein kinase (EC 2.7.11.24)	Pkinase; Pkinase_Tyr
	AS4DTRINITY_DN8188_c0_g1_i2	AT1G08260|AT1G08260.1	DNA polymerase epsilon catalytic subunit (EC 2.7.7.7)	DNA_pol_B; DNA_pol_B_exo1; DNA_pol_B_exo2; DUF1744
	BB4DTRINITY_DN13385_c3_g2_i2	AT1G30490|AT1G30490.1	Uncharacterized protein	Homeobox; MEKHLA; START
	BS4DTRINITY_DN10864_c0_g3_i1	Vigun05g126900	Uncharacterized protein	ANAPC3; TPR_1; TPR_12; TPR2; TPR
	BS7DTRINITY_DN12088_c3_g6_i6	AT1G63700|AT1G63700.1	Protein kinase domain-containing protein;mitogen-activated protein kinase kinase kinase YODA-like isoform X2;hypothetical protein	Kinase-like; Pkinase; Pkinase_Tyr
	CB4DTRINITY_DN13007_c0_g1_i1	Vigun03g187300	BZIP domain-containing protein;ABSCISIC ACID-INSENSITIVE 5-like protein 7;hypothetical protein	bZIP_1; bZIP_2
	CS4DTRINITY_DN10654_c0_g1_i1	AT1G08260|AT1G08260.1	DNA polymerase epsilon catalytic subunit (EC 2.7.7.7)	DNA_pol_B; DNA_pol_B_exo1; DNA_pol_B_exo2; DUF1744
	CS4DTRINITY_DN11557_c0_g1_i4	AT5G10330|AT5G10330.1; AT5G10330.2;AT5G10330.3	Histidinol-phosphate aminotransferase	Alliinase_C; Aminotran_1_2
	CS4DTRINITY_DN11622_c2_g5_i1	AT5G15800|AT5G15800.2; AT5G15800.1	Uncharacterized protein;agamous-like MADS-box protein AGL6;hypothetical protein	K-box; SRF-TF
	CS4DTRINITY_DN12139_c1_g3_i1	AT1G30490|AT1G30490.1	Uncharacterized protein	Homeobox; MEKHLA; START

**Days to flowering**	AB4DTRINITY_DN7670_c0_g1_i4	LOC101501589	transcription factor PIF3-like isoform X2	HLH
	AB7DTRINITY_DN30303_c0_g10_i1	LOC101496618	UMP pyrophosphorylase;uracil phosphoribosyltransferase isoform X1;hypothetical protein	UPRTase
	AB7DTRINITY_DN30388_c1_g5_i1	LOC101503257	Cryptochrome-1	Cryptochrome_C; DNA_photolyase; FAD_binding
	AS4DTRINITY_DN12051_c1_g15_i4	LOC101511540	LOW QUALITY PROTEIN: protein GIGANTEA-like	Aldo_ket_red
	AS4DTRINITY_DN6224_c0_g1_i2	LOC101496618	Retrovirus-related Pol polyprotein from transposon TNT 1-94	RVT_2
	AS7DTRINITY_DN11110_c4_g6_i1	LOC101503257	Photolyase/cryptochrome alpha/beta domain-containing protein	Cryptochrome_C; DNA_photolyase; FAD_binding
	AS7DTRINITY_DN20408_c0_g1_i1	LOC101489582	Uncharacterized protein	CCT
	BB4DTRINITY_DN13521_c0_g1_i10	LOC101508547	protein LEO1 homolog	Leo1
	BB7DTRINITY_DN16202_c0_g2_i1	LOC101497653	ethylene-responsive transcription factor RAP2-7-like isoform X2	AP2
	BS4DTRINITY_DN20963_c0_g1_i1	LOC101509277	Fe2OG dioxygenase domain-containing protein	2OG-FeII_Oxy
	BS7D1TRINITY_DN10733_c0_g3_i2	LOC101497653	Uncharacterized protein	AP2
	BS7DTRINITY_DN8317_c0_g2_i1	LOC101506511	Phytochrome	PAS
	CB4DTRINITY_DN11833_c0_g3_i1	LOC101503257	Photolyase/cryptochrome alpha/beta domain-containing protein	Cryptochrome_C; FAD_binding
	CB7DTRINITY_DN17668_c0_g3_i3	LOC101496618	protein FLOWERING LOCUS T-like	PBP
	CS4DTRINITY_DN10983_c0_g1_i2	LOC101514145	two-component response regulator-like APRR1 isoform X4	CCT; Response_reg
	CS4DTRINITY_DN11159_c2_g4_i2	LOC101503257	Photolyase/cryptochrome alpha/beta domain-containing protein	Cryptochrome_C; DNA_photolyase; FAD_binding
**Seed weight**	AB4DTRINITY_DN10404_c2_g6_i1	Phvul.008G120700; Phvul.010G010200	Expansin	DPBB_1
	AB4DTRINITY_DN10598_c3_g1_i1	Phvul.006G077200	Expansin	DPBB_1; Pollen_allerg_1
	AB4DTRINITY_DN16153_c0_g1_i2	Phvul.011G014000	Cytokinin dehydrogenase;cytokinin dehydrogenase 6-like;hypothetical protein	Cytokin-bind; FAD_binding
	AB7DTRINITY_DN30367_c2_g3_i1	Phvul.008G120700; Phvul.010G010200	expansin-A4-like	DPBB_1; Pollen_allerg_1
	AB7DTRINITY_DN33078_c0_g1_i1	AT2G37640	Expansin	DPBB_1; Pollen_allerg_1
	AS4DTRINITY_DN1065_c0_g1_i1	Phvul.003G099000;Phvul.006G159300	HPt domain-containing protein	Hpt
	AS4DTRINITY_DN11254_c0_g6_i1	Phvul.003G253100	Uncharacterized protein;cytochrome P450 90B1-like;hypothetical protein	p450

	AS4DTRINITY_DN11422_c1_g2_i1	Phvul.003G196600	Uncharacterized protein	E2F_TDP
	AS4DTRINITY_DN3962_c0_g1_i1	Phvul.001G261500	Uncharacterized protein	p450
	AS7DTRINITY_DN8390_c0_g1_i1	Phvul.003G196500	Response regulatory domain-containing protein	Response_reg
	BB4DTRINITY_DN13171_c6_g7_i1	Phvul.008G120700; Phvul.010G010200	Expansin	DPBB_1; Pollen_allerg_1
	BB7DTRINITY_DN16316_c5_g6_i2	Phvul.008G120700; Phvul.010G010200	Expansin	DPBB_1; Pollen_allerg_1
	BS4DTRINITY_DN10952_c0_g1_i3	Phvul.003G196600	Uncharacterized protein	E2F_TDP
	BS4DTRINITY_DN12034_c2_g3_i1	Phvul.003G253100	Uncharacterized protein	p450
	BS4DTRINITY_DN12381_c0_g4_i1	Phvul.006G077200	Expansin	Pollen_allerg_1
	BS4DTRINITY_DN13044_c1_g3_i3	Phvul.008G120700; Phvul.010G010200	Expansin	DPBB_1; Pollen_allerg_1
	BS7D1TRINITY_DN9417_c0_g2_i1	Phvul.003G099000;Phvul.006G159300	HPt domain-containing protein	Hpt
	CB4DTRINITY_DN14045_c9_g6_i1	Phvul.008G120700; Phvul.010G010200	Expansin	DPBB_1; Pollen_allerg_1
	CB4DTRINITY_DN14188_c1_g8_i2	Phvul.003G253100	Uncharacterized protein	p450
	CB4DTRINITY_DN14247_c0_g4_i1	Phvul.006G077200	Expansin;expansin-A8-like;hypothetical protein	DPBB_1; Pollen_allerg_1
	CB7DTRINITY_DN15484_c0_g2_i1	Phvul.003G196600	Uncharacterized protein	E2F_TDP
	CB7DTRINITY_DN35628_c0_g1_i1	Phvul.003G099000;Phvul.006G159300	HPt domain-containing protein	Hpt
	CS4DTRINITY_DN10656_c0_g1_i5	Phvul.003G196600	E2F_TDP domain-containing protein	E2F_TDP
	CS4DTRINITY_DN11417_c0_g2_i1	Phvul.008G120700; Phvul.010G010200	Expansin	DPBB_1; Pollen_allerg_1
	CS4DTRINITY_DN12484_c15_g1_i1	Phvul.006G077200	Expansin;expansin-A4-like;hypothetical protein	DPBB_1; Pollen_allerg_1
	CS4DTRINITY_DN4103_c0_g1_i1	Phvul.004G064600_CYP735A1	cytokinin hydroxylase-like	p450
	CS4DTRINITY_DN5774_c0_g1_i1	Phvul.011G014000	Cytokinin dehydrogenase;cytokinin dehydrogenase 6-like;hypothetical protein	Cytokin-bind; FAD_binding
	CS4DTRINITY_DN9587_c0_g1_i4	Phvul.003G253100	cytochrome P450 90B1 isoform X2	p450
**Pod Shattering**	AB4DTRINITY_DN10692_c1_g7_i3	Phvul.009G138600; Phvul.002G266400	probable WRKY transcription factor 13 isoform X2	WRKY
	AB4DTRINITY_DN11033_c2_g19_i1	Phvul.002G076700	metalloendoproteinase 1-like	Peptidase_M10; PG_binding_1
	AB4DTRINITY_DN11168_c0_g3_i2	Phvul.002G188600;Phvul.009G242700	cellulose synthase A catalytic subunit 4 [UDP-forming]-like	zf-RING; zf-UDP

	AB4DTRINITY_DN11214_c0_g9_i1	Phvul.002G110900;Phvul.003G260500;Phvul.005G044600	NAC domain-containing protein;NAC domain-containing protein 37-like isoform X1;NAC domain-containing protein 37-like isoform X1;hypothetical protein	NAM
	AB4DTRINITY_DN21800_c0_g1_i1	Phvul.002G188600;Phvul.009G205100	Cellulose synthase (EC 2.4.1.12)	Cellulose_synt
	AB4DTRINITY_DN5619_c0_g1_i3	Phvul.011G212000	Uncharacterized protein	Myb_DNA-binding; MYB_DNA_bind
	AB7DTRINITY_DN30825_c4_g1_i2	Phvul.010G071300	Uncharacterized protein	zf-C2H2
	AB7DTRINITY_DN30910_c4_g2_i2	Phvul.009G205100;Phvul.002G188600	Cellulose synthase (EC 2.4.1.12)	Cellulose_synt; zf-RING; zf-UDP
	AB7DTRINITY_DN30942_c0_g2_i7	Phvul.004G093300;Phvul.005G022100;Phvul.009G205100	Cellulose synthase (EC 2.4.1.12)	Cellulose_synt; zf-RING; zf-UDP
	AB7DTRINITY_DN31057_c0_g11_i1	Phvul.004G093300	Cellulose synthase (EC 2.4.1.12)	Cellulose_synt
	AB7DTRINITY_DN31083_c0_g1_i3	Phvul.003G154600;Phvul.009G205100;Phvul.009G242700	Cellulose synthase (EC 2.4.1.12)	Cellulose_synt; FYVE2; Glyco_trans_2; zf-RING; zf-UDP
	AB7DTRINITY_DN31083_c0_g4_i1	Phvul.009G242700;Phvul.002G188600	Non-specific serine/threonine protein kinase;CBL-interacting serine/threonine-protein kinase 8-like isoform X1;hypothetical protein	NAF; Pkinase; Pkinase_Tyr; zf-RING; zf-UDP
	AB7DTRINITY_DN31083_c0_g5_i7	Phvul.009G242700;Phvul.002G188600;Phvul.007G081700;Phvul.011G211500	Cellulose synthase (EC 2.4.1.12)	Cellulose_synt; Glyco_trans_2
	AB7DTRINITY_DN31083_c0_g6_i2	Phvul.009G094200;Phvul.009G205100;Phvul.002G188600	Cellulose synthase (EC 2.4.1.12)	Cellulose_synt; Glyco_trans_2
	AB7DTRINITY_DN31344_c6_g1_i6	Phvul.009G138600;Phvul.002G266400	WRKY domain-containing protein	WRKY
	AS4DTRINITY_DN10999_c2_g7_i4	Phvul.009G138600;Phvul.002G266400	Putative WRKY transcription factor 13 (Fragment)	WRKY
	AS4DTRINITY_DN11024_c1_g1_i1	Phvul.011G005700	NAC domain-containing protein	NAM
	AS4DTRINITY_DN11137_c2_g12_i1	Phvul.009G090100;Phvul.002G240200	Cellulose synthase (EC 2.4.1.12)	Cellulose_synt
	AS4DTRINITY_DN11563_c3_g14_i1	Phvul.002G188600;Phvul.009G242700;Phvul.009G205100	Cellulose synthase (EC 2.4.1.12)	Cellulose_synt; Glyco_trans_2
	AS4DTRINITY_DN11563_c3_g5_i6	Phvul.004G093300	Cellulose synthase (EC 2.4.1.12)	Cellulose_synt
	AS7DTRINITY_DN10787_c3_g4_i2	Phvul.009G090100;Phvul.002G240200	Cellulose synthase (EC 2.4.1.12)	Cellulose_synt; Glyco_trans_2; zf-UDP

	AS7DTRINITY_DN10787_c3_g5_i1	Phvul.009G205100;Phvul.003G154600;Phvul.005G022100;Phvul.011G211500;Phvul.002G240200;Phvul.011G020100;	Cellulose synthase;cellulose synthase A catalytic subunit 3 [UDP-forming];cellulose synthase A catalytic subunit 3 [UDP-forming];cellulose synthase A catalytic subunit 3 [UDP-forming];hypothetical protein	Cellulose_synt; Glyco_trans_2; zf-RING
	AS7DTRINITY_DN6199_c0_g1_i1	Phvul.008G211900	protein FAM91A1	FAM91_C
	BB4DTRINITY_DN12591_c8_g1_i7	Phvul.009G138600;Phvul.002G266400	WRKY domain-containing protein	WRKY
	BB4DTRINITY_DN32604_c0_g1_i1	Phvul.002G076600_PGDB	ZnMc domain-containing protein	Peptidase_M10; PG_binding_1
	BB4DTRINITY_DN3569_c0_g1_i1	Phvul.007G081700;Phvul.004G093300	Cellulose synthase (EC 2.4.1.12)	FYVE_2; zf-RING; zf-UDP
	BS4DTRINITY_DN12471_c2_g1_i5	Phvul.011G211500;Phvul.005G022100;Phvul.007G081700	Cellulose synthase (EC 2.4.1.12)	Cellulose_synt; zf-UDP
	BS4DTRINITY_DN12817_c1_g3_i1	Phvul.002G188600;Phvul.009G242700	Cellulose synthase (EC 2.4.1.12)	Cellulose_synt; Glyco_trans_2
	BS4DTRINITY_DN12863_c0_g1_i1	Vigung03g306000.1;Phvul.003G217600;Phvul.002G061000;Phvul.011G005700	NAC domain-containing protein	NAM
	BS7D1TRINITY_DN10602_c3_g8_i2	Phvul.010G118700	NAC domain-containing protein	Cupin_1; NAM
	BS7D1TRINITY_DN10624_c1_g10_i2	Phvul.009G205100;Phvul.003G154600	cellulose synthase A catalytic subunit 7 [UDP-forming]	Cellulose_synt
	BS7D1TRINITY_DN10624_c1_g9_i1	Phvul.009G242700;Phvul.009G094200;Phvul.002G188600;	Cellulose synthase (EC 2.4.1.12)	Cellulose_synt; Glyco_trans_2; zf-RING; zf-UDP
	BS7D1TRINITY_DN10934_c0_g3_i2	Phvul.009G205100;Phvul.003G154600;Phvul.009G242700	Cellulose synthase (EC 2.4.1.12)	Cellulose_synt; zf-RING; zf-UDP
	BS7D1TRINITY_DN7837_c1_g2_i1	Vigun03g180302600.1;Phvul.003G221000;Phvul.002G058900	Uncharacterized protein	zf-C2H2; zf-C2H2_jaz
	BS7DTRINITY_DN10365_c0_g1_i2	Phvul.008G048100;Phvul.010G046500;Medtr8g66007270	WRKY domain-containing protein	WRKY
	BS7DTRINITY_DN11845_c1_g1_i4	Phvul.009G090100;Phvul.002G240200	Cellulose synthase (EC 2.4.1.12)	Cellulose_synt; Glyco_trans_2
	BS7DTRINITY_DN11845_c1_g7_i1	Phvul.009G205100;Phvul.003G154600;Phvul.002G240	Cellulose synthase (EC 2.4.1.12)	Cellulose_synt; Glyco_trans_2; zf-RING; zf-UDP

		200;Phvul.009G242700;Phvul.011G020100		
	CB4DTRINITY_DN13190_c1_g1_i4	Vigun07g046100	AP2/ERF domain-containing protein	AP2
	CB4DTRINITY_DN17299_c0_g1_i1	Phvul.003G154600;Phvul.009G205100	Cellulose synthase (EC 2.4.1.12)	zf-UDP
	CB4DTRINITY_DN18196_c0_g1_i1	Phvul.009G070800	Uncharacterized protein	zf-C2H2
	CB4DTRINITY_DN23701_c0_g1_i1	Phvul.002G266400	WRKY domain-containing protein	WRKY
	CB7DTRINITY_DN10850_c0_g2_i1	Vigun03g180302600.1;Phvul.003G221000;Phvul.002G058900	Uncharacterized protein	zf-C2H2; zf-C2H2_jaz
	CB7DTRINITY_DN16459_c1_g1_i1	Phvul.008G048100;Phvul.010G046500;Medtr8g66007270	WRKY domain-containing protein	WRKY
	CB7DTRINITY_DN16958_c0_g5_i1	Phvul.008G211900	protein FAM91A1-like isoform X2	FAM91_C
	CB7DTRINITY_DN17428_c3_g14_i3	Phvul.009G242700;Phvul.002G188600	Cellulose synthase (EC 2.4.1.12)	Cellulose_synt; Glyco_trans_2
	CB7DTRINITY_DN17428_c3_g5_i3	Phvul.003G154600;Phvul.009G205100	Cellulose synthase (EC 2.4.1.12)	Cellulose_synt; Glyco_trans_2; zf-RING; zf-UDP
	CB7DTRINITY_DN21024_c0_g1_i1	Vigun07g046100	AP2/ERF domain-containing protein	AP2
	CS4DTRINITY_DN11295_c0_g1_i6	Phvul.009G138600;Phvul.002G266400	WRKY domain-containing protein	WRKY
	CS4DTRINITY_DN11355_c0_g10_i1	Phvul.002G188600;Phvul.009G242700	Cellulose synthase (EC 2.4.1.12)	Cellulose_synt
	CS4DTRINITY_DN11441_c0_g2_i1	Phvul.004G093300	serine/threonine-protein phosphatase 7 long form homolog	Cellulose_synt
	CS4DTRINITY_DN12002_c0_g6_i5	Phvul.002G076700	metalloendoproteinase 1-like	Peptidase_M10; PG_binding_1
	CS4DTRINITY_DN12033_c3_g1_i2	Vigung03g306000.1;Phvul.003G217600;Phvul.002G061000	NAC domain-containing protein	NAM
	CS4DTRINITY_DN23449_c0_g1_i1	Phvul.010G046500;Medtr8g66007270;Phvul.008G048100	WRKY domain-containing protein;probable WRKY transcription factor 12;hypothetical protein	WRKY
**Pod length**	CB4DTRINITY_DN610_c0_g1_i1	Phvul.001G173700|Phvul.001G173700.1	Uncharacterized protein	WRKY
	CB7DTRINITY_DN16977_c3_g12_i1	Glyma.07G134800|Glyma.07G134800.1	Auxin response factor	AUX_IAA
	CS4DTRINITY_DN10719_c0_g1_i4	Glyma.07G134800|Glyma.07G134800.1	Auxin response factor	Auxin_resp; AUXIAA; B3

	CS4DTRINITY_DN5469_c0_g1_i1	Phvul.001G173700|Phvul.001G173700.1	Uncharacterized protein	WRKY
	CS4DTRINITY_DN5646_c0_g1_i1	Glyma.16G129600|Glyma.16G129600.1	Uncharacterized protein	Auxin_inducible
